# Silicon-Solubilizing Media and Its Implication for Characterization of Bacteria to Mitigate Biotic Stress

**DOI:** 10.3389/fpls.2020.00028

**Published:** 2020-02-28

**Authors:** Vidisha Bist, Abhishek Niranjan, Manish Ranjan, Alok Lehri, Karishma Seem, Suchi Srivastava

**Affiliations:** ^1^ Division of Microbial Technology, CSIR-National Botanical Research Institute, Lucknow, India; ^2^ Academy of Scientific and Innovative Research (AcSIR), Ghaziabad, India

**Keywords:** silicon, silicon-solubilizing bacteria, silicon fertilization, acidic phosphatase, feldspar, rice sheath blight, *Rhizoctonia solani*, *Bacillus amyloliquefaciens*

## Abstract

Silicon (Si), the second most abundant element on earth, remains unavailable for plants' uptake due to its poor solubility. Microbial interventions to convert it in soluble forms are well documented. However, studies on discrimination of Si and P solubilizing microbes due to common estimation method and sharing of solubilization mechanism are still obscure. A defined differential media, i.e. silicon-solubilizing media (NBRISSM) is developed to screen Si solubilizers. NBRISN13 (*Bacillus amyloliquefaciens*), a Si solubilizer, exhibiting antagonistic property against *Rhizoctonia solani*, was further validated for disease resistance. The key finding of the work is that NBRISSM is a novel differential media for screening Si solubilizers, distinct from P solubilizers. Dominance of *Pseudomonas* and *Bacillus* spp. for the function of Si solubilization was observed during diversity analysis of Si solubilizers isolated from different rhizospheres. *Sphingobacterium* sp., a different strain has been identified for silicon solubilization other than *Pseudomonas* and *Bacillus* sp. Role of acidic phosphatase during Si solubilization has been firstly reported in our study in addition to other pH dependent phenomenon. Study also showed the combinatorial effect of feldspar and NBRISN13 on elicited immune response through (i) increased Si uptake, (ii) reduced disease severity, (iii) modulation of cell wall degrading and antioxidative enzyme activities, and (iv) induced defense responsive gene expression.

**Graphical Abstract:**
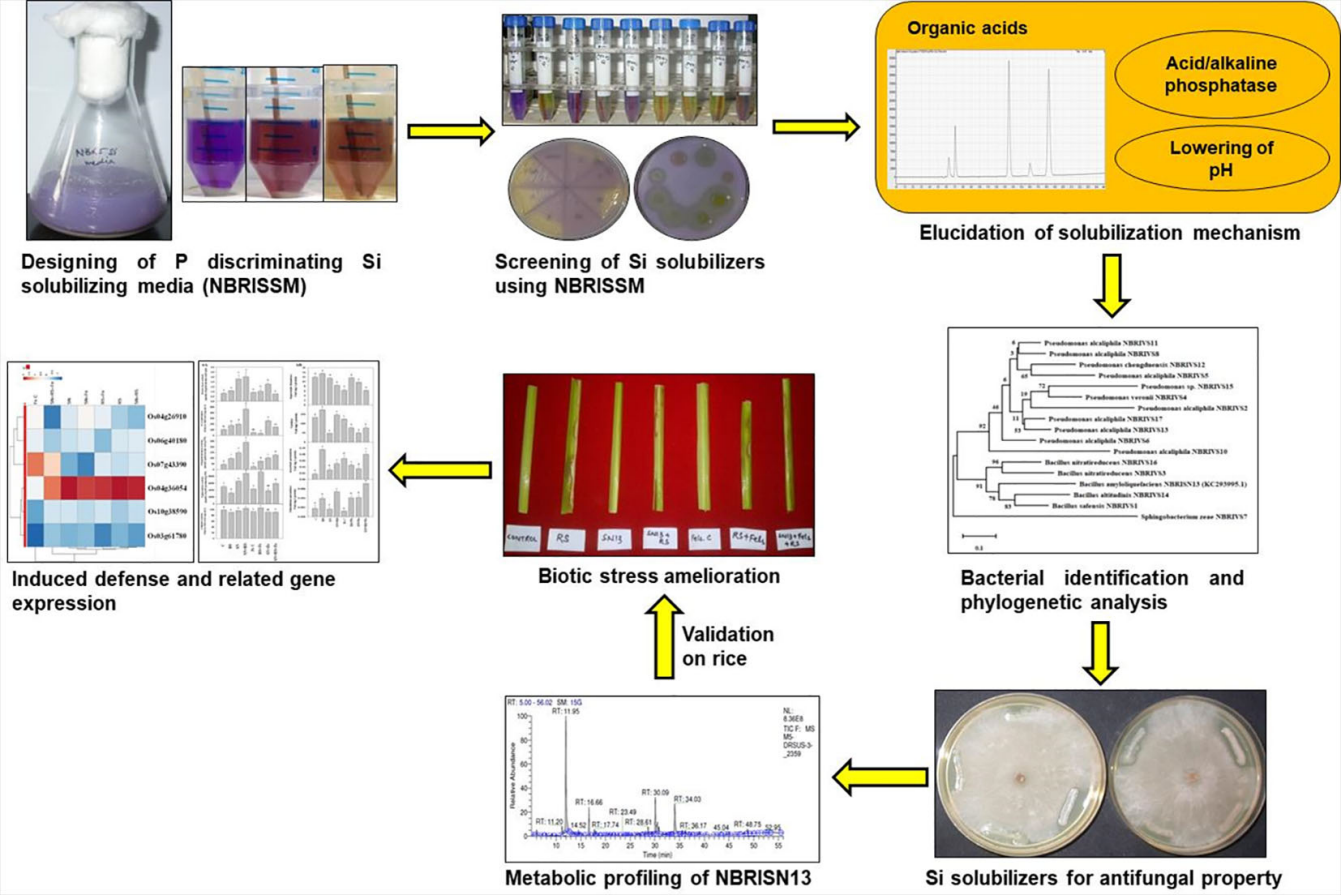


## Introduction

Silicon (Si), the second most abundant element (27%), usually occurs in the unavailable forms of silicates (SiO_3_) of aluminum, magnesium, calcium, sodium, potassium, or iron. It's accessibility to the plant roots is generally governed by chemical and biological reactions of soil. Plants assimilate Si as soluble monosilicic acid resulting in strengthening of cell wall through various mechanisms (Pilon-Smits et al., 2009; Malhotra et al., 2016). Strengthened cell wall due to higher accumulation of silicon is known to improve plant resistance to diseases, insect attack, and adverse climatic conditions in various plant species like rice, oat, barley, wheat, cucumber,and sugarcane (Liang et al., 2003; Fauteux et al., 2005; Cai et al., 2008; Yavaş and Aydın, 2017).

Rice exhibits higher silicon (Si) uptake varying from 0.1% to 10% dry weight of shoot, making the cell walls thick and rigid, in-turn playing beneficial role in plant growth and development (Fleck et al., 2015; Bokor et al., 2017; Głazowska et al., 2018). Improved nutrient availability, increased abiotic and biotic stress tolerance, and enhanced fertilizer use efficiency are some known beneficial effects of silicon (Agostinho et al., 2017; Rao et al., 2017; Lee et al., 2019). Repeated cropping reduces the level of plant available silicon, thus enforcing the application of silicon fertilizers in forms of fine silica, sodium/calcium/potassium silicate, along with other chemical fertilizers (Datnoff and Rodrigues, 2005).

Microorganisms are known to play major role in dissolution of minerals like silicates and phosphates (Chen et al., 2016b; Lee et al., 2019). Several beneficial microbes have been reported for their positive impacts on plant under different stress conditions through better uptake of these minerals (Rogers and Bennett, 2004; Lee et al., 2019). Solubilization of both insoluble silicon and phosphate due to organic acid production by microbes is known to enhance their availability to plants (Ameen et al., 2019). Various studies have reported weathering of silicates by bacteria for its dissolution to make it available to the plants (Chandrakala et al., 2019). Different groups have developed the liquid screening media containing different silicon sources *viz.* feldspar, muscovite, biotite, and magnesium trisilicate with soluble phosphate sources (di-potassium hydrogen phosphate; K_2_HPO_4_ and di-sodium hydrogen phosphate; Na_2_HPO_4_); and agar based media containing glucose and magnesium trisilicate as sole source of nutrition (Sheng et al., 2008; Kang et al., 2017; Vasanthi et al., 2018). Number of bacterial strains of genus *Bacillus, Pseudomonas, Proteus, Rhizobia, Burkholderia*, and *Enterobacter* are known to release silicon from silicates and promote plant growth (Meena et al., 2014a; Wang et al., 2015; Kang et al., 2017; Kumawat et al., 2017; Chandrakala et al., 2019; Lee et al., 2019). However, due to the common mechanism of solubilization (organic acid production) and quantification method (molybdenum blue method) for both phosphate (P, Fiske and Subbarow, 1925) and silicon (Si, Elliott and Synder, 1991) there is unavailability of a concrete silicon-solubilizing media.

Number of phosphate assimilating microbe based bio-fertilizers are used for phosphate availability in different crop system (Alori et al., 2017; Mohammadi, 2017). Likewise, there is a need of microbial intervention to facilitate silicon availability in soil-plant system for development of silicon-solubilizing microbe based bio-fertilizers. Therefore, in order to harness the microbial potential, unearth the mechanism of silicon solubilization and its role in biotic stress tolerance, the present study focuses on (i) designing of Si-solubilizing P discriminating media (NBRISSM) for efficient screening of Si solubilizers with minimum P interference, (ii) elucidation of mechanism for Si solubilization through organic acid profiling and its correlation with acidic phosphatase activity, (iii) its implication for enhancement of biotic stress mitigation in rice against *Rhizoctonia solani* (RS)- the major pathogen of rice sheath blight disease causing 25% to 40% yield loss (Lee and Rush, 1983; Srivastava et al., 2016).

## Materials and Methods

### Bacterial Strains and Culture Conditions

Considering the common mechanism of phosphate (P) and silicon (Si) solubilization, National Botanical Research Institute's phosphate growth medium (NBRIP; screening media for P solubilizers, Nautiyal, 1999) was used as basal medium to develop the screening medium for Si solubilizers (Fiske and Subbarow, 1925; Chen et al., 2016a; Vasanthi et al., 2018). Abiotic stress tolerant *Bacillus amyloliquefaciens* (Acc. No KC293995.1, B2; NBRISN13, Nautiyal et al., 2013a) with antagonistic activity against RS (Srivastava et al., 2016); and *Bacillus safensis* (Acc. No MN715812.1, B1; NBRIVS1) were used as model strains for development of media. Cultures were grown in 150 ml polypropylene Erlenmeyer flasks (Vasanthi et al., 2018) containing 50 ml of medium. Bacterial inoculations were made @ 1% (approximately 1–2 × 10^9^ cfu ml^−1^) and the uninoculated medium served as control followed by their incubation at 28°C, 180 rpm on a New Brunswick Scientific, USA, Innova Model 4230 refrigerated incubator shaker for 10 days. The cultures were sampled at 1st, 3rd, 5th, 7th, and 10th day post inoculation for the estimation of solubilized Si in triplicates. All the experiments were repeated for three times. For qualitative screening, appearance of change in color after 3 to 5 days of bacterial inoculation was considered as indicator of the Si-solubilizing activity.

### Quantitative Estimation of Solubilized Si

Solubilized Si by various bacterial strains grown in different silicon media was quantified by the modified method of Elliott and Snyder (1991) in triplicates. In brief, culture supernatant was mixed with three volumes of 2.5% boric acid and equal volume of 5.4% ammonium molybdate followed by 5-min incubation. Further, 20% tartaric acid and 0.5% ascorbic acid sodium salt was added in the total volume of 1ml. The solution was vortexed and the absorbance was measured at 650 nm spectrophotometrically. Quantity of solubilized Si was estimated against the SiO_2_ standard. Silicon estimation in soil was performed by extraction of available Si by acetate buffer method in duplicate (Abe et al., 2016). In brief, 5 g soil was mixed with 50 ml of 1 mol/L sodium acetate (pH = 4.0) and incubated at 40°C for 5 h. The soil solution was further used for the estimation of Si, using the modified molybdenum blue method in triplicate (Elliott and Synder, 1991).

### Development of NBRISSM Medium

In order to develop a defined medium for screening of Si-solubilizing microbes, insoluble sources of silicon *viz*, magnesium trisilicate, talc, and feldspar were amended to the NBRIP medium. Organic acid production being the common mechanism for P and Si solubilization raise the need to make the appropriate choice of phosphate source. Therefore, tri-Calcium phosphate (TCP) was replaced by different P sources such as hydroxyapatite (inorganic) and sodium phytate (organic), whereas, potassium di-hydrogen phosphate (KH_2_PO_4_) served as positive control. After the selection of silicon and phosphate sources selection of appropriate carbon (C) source was targeted. Different nine primary and secondary carbon sources *viz*. glucose, sucrose, lactose, mannitol, L-arabinose, sorbitol, sodium citrate, sodium acetate, and sodium benzoate were tested by replacing glucose from NBRIP medium for better silicon solubilization. Ammonium sulfate, the nitrogen source of the NBRIP medium was replaced by ammonium nitrate, ammonium sulfate, ammonium chloride, ammonium ferric citrate, ammonium tartrate, and aluminum ammonium sulfate. For selection of better salt combinations to be used in medium, 15 different salts of magnesium, potassium, calcium, and sodium were checked for better Si solubilization. In order to define the medium composition, the quantity of all components was determined after taking one higher and one lower concentration of that component used in NBRIP medium to get maximum Si and minimum P interference. The basal medium NBRIP contained l^−1^: 10 g glucose; 5 g Ca_3_(PO_4_)_2_; 5 g MgCl_2_.6H_2_O; 0.25 g MgSO_4_.7H_2_O; 0.2 g KCl; and 0.1 g (NH_4_)_2_SO_4,_ whereas, the developed silicon-solubilizing media (NBRISSM) contains l^−1^: 2.5 g glucose; 2.5 g hydroxyapatite; 1.25 g MgNO_3_; 1.25 g CaCl_2_; 0.1 g (NH_4_)_2_SO_4_; 0.1 g Mg_2_O_8_Si_3_; 0.025g. The above discussed amendments enlisted in **Supplementary Tables (S1–S3)**, *viz*. various silicon, phosphate, carbon, nitrogen, and salt sources were repeated thrice to design NBRISSM medium. Qualitative screening was performed as color change from blue to purple/yellow which persists until fifth or seventh day of inoculation, where bromo cresol purple (BCP) was used as pH indicator. Different concentrations of BCP, i.e. 0.00125%, 0.0025%, 0.005% and 0.01% was used and spectrophotometrically scanned to determine the shift in optical density for qualitative differentiation of silicon-solubilizing bacterial strains. The pH of the medium was adjusted to 7.0 before autoclaving.

### Comparison of P Discriminating Si Media (NBRISSM) With Existing Media

NBRISSM medium (A) was compared with the other media reported earlier for Si solubilization, i.e. medium (B) g/l: Glucose 10 g; NH_4_SO_4_ 1.0 g; KCl_2_ 0.2 g; MgSO_4_ 0.2 g; K_2_HPO_4_ 0.1 g; pH 7.0 to 7.2 (Vasanthi et al., 2018), medium (C) g/l: 1% sucrose, 0.1% (NH_4_)_2_SO_4_, 0.05% Na_2_HPO_4_, 0.05% MgSO_4_, 0.01% NaCl, 0.05% yeast extract, 1% feldspar powder (75–150 mm), pH 7.2 (Sheng et al., 2008); medium (D) i.e. NBRIP (basal medium) (g/l): glucose, 10 g: Ca_3_(PO_4_)_2_, 5 g; MgCl_2_.6H_2_O, 5 g; MgSO_4_.7H_2_O, 0.25 g; KCl, 0.2 g; and (NH_4_)_2_SO_4_, 0.1 g (Nautiyal, 1999). Selected bacterial strains (B1- N.H and B2- N.S) were grown in these media in triplicates at 28°C for 10 days and media supernatant was used for quantitative assay of solubilized silicon in terms of silicic acid (the plant available form) (Elliott and Snyder, 1991) and P (Fiske and Subbarow, 1925).

Quantitative estimation of P was performed in the culture supernatant grown in NBRISSM by the method of Fiske and Subbarow (1925). In brief, the culture supernatant is mixed with ammonium molybdate (1:5) and coloring reagent (alpha amino naphthol sulfonic acid) for the spectrophotometric determination of P at 660 nm in triplicates.

### Screening of Silicon Solubilizing Microbe Using NBRISSM-BCP Media

Bacterial strains belonging to different locations and rhizosphere were grown in NBRISS-BCP medium and qualitative screening based on color change from purple toward yellow was performed. Quantitative estimation of solubilized Si in the culture supernatant grown in NBRISSM media was performed by the modified method of Elliott and Synder (1991) as described above.

### Organic Acid Quantification

Organic acid produced during the process of Si solubilization was estimated to identify marker organic acids. Microbes with different efficacies of Si and P solubilization along with best Si solubilizers screened with the developed NBRISSM media were used for organic acid profiling using HPLC (Shimadzu, Japan) comprising PDA SPD M 20 A system LC-20AD dual pump system, and SIL-20 AC auto injector (with cooler) furnished with a 20 µL sample loop. Compounds were separated on column with 250 × 4.6 mm, i.d. (inside diameter) and 5 µm pore size, Shimadzu RP-C18 column protected by guard column containing the same packing. The mobile phase of 0.01mol/L sulfuric acid was used for the elution at the flow rate of 0.5 ml/min (Wang et al., 2014). In brief, the culture supernatant of different microbial cultures grown in NBRISSM medium after 5^th^ day of inoculation was injected thrice with 20 µl sample loop and run for 25 min. Data was integrated by Shimadzu Lab solution software with the detection of peaks at 510 nm and results were obtained by comparison with standards. Plain mobile phase was used as control for identification of blank peaks.

### Acidic/Alkaline Phosphatase Activity

In order to correlate Si solubilization with other pH dependent phenomenon, acid and alkaline phosphatase activity was assayed in bacterial culture grown in NBRISSM containing hydroxyapatite as P source (Du Plessis et al., 2007). In brief, the cultures were lysed using toluene and incubated for 2 h at 28°C. In a 2.0 ml of reaction mixture, 1.5 ml aqueous layer, 0.2 ml of 0.05 M acetate buffer (pH 5.0) for acidic and 1 M Tris (pH 8.0) for alkaline phosphatase and 0.3 ml of pNPP (10 mg/ml, prepared in respective buffers) as substrate was added. The reaction mixture was incubated at 37°C for 45 min. The reaction was stopped by adding 1 ml of 3M NaOH. The activity [liberated p-nitrophenol (pNP)] was measured spectrophotometrically at 410nm in triplicates.

### Systematics of Best Si Solubilizers

Systematics of different Si solubilizers was performed through 16S rDNA sequencing as described earlier (Nautiyal et al., 2013a). In brief 16S rRNA gene was amplified using forward 27F (5′-AGAGTTTGATCCTGGCTCAG-3′) and reverse 1492R (5′-AAGGAGGTGATCCAGCCGCA-3′) primers under standard PCR conditions (Initial denaturation at 94°C for 5 min, 30 cycles of denaturation at 94°C for 1 min, annealing at 50°C for 40 s, extension at 72°C for 90 s, and final extension at 72°C for 7 min) (Weisburg et al., 1991; Cui et al., 2001; Nair et al., 2018). Purified PCR product (~1.4 kb) was sequenced at Central Instrument Facility at National Botanical Research Institute, Lucknow using ABI 3730XL capillary DNA sequencer (50 cm capillary). The obtained partial 16S rRNA gene sequences were aligned and identified using BLAST analysis. The sequences were submitted to GenBank for the generation of accession numbers and the phylogenetic tree was constructed by MEGA-X software. MEGA based phylogenetic tree was constructed using Evolutionary analysis by Maximum Likelihood method and Kimura 2-parameter model by applying Neighbor-Join and BioNJ algorithms to a matrix of pairwise distances estimated using the Maximum Composite Likelihood (MCL) approach, and then selecting the topology with superior log likelihood value. The resulting tree was evaluated by 1,000 bootstrap replicates, considering complete sequence sites with Type strains mentioned in **Figure 6** (Kumar et al., 2018).

### Screening of Biocontrol Agents for Antifungal Property

Different Si-solubilizing strains were screened for their antifungal property against RS using dual culture plate method. The bacterial strains were streaked at the edges of media plates [Nutrient agar (NA) + Potato Dextrose agar (PDA); 1:1] with 6mm circular discs of fungal mat placed at the center of the plate. The plates were incubated at 28˚C until the control plate attained the maximum growth. Control setup was the fungus (RS) alone.

### Metabolite Extraction

Best selected bacterial strain with already proven biocontrol activity against *R. solani* was chosen for metabolic profiling. Ethyl acetate extract of the bacterial culture grown in nutrient broth was prepared through solvent extraction method. In brief 72h grown cultures of NBRISN13 and RS (alone and simultaneous inoculation) were extracted with 1/3^rd^ volume of ethyl acetate. The process was repeated for three times and the pooled ethyl acetate fraction was dried and converted to methyl esters before GC-MS analysis. In brief, the extracted metabolite was derivatized by dissolving 4-5 mg of extract in 50 μL of methoxyamine-HCL solution prepared in GC grade pyridine (20 mg/mL). Reaction mixture was subjected to shaking conditions for 2 h at 40°C using thermo mixer Comfort (Eppendorf India Ltd.) with further addition of 70 μL of N-Methyl- N-(trimethylsilyl) trifluoroacetamide. Again the shaking of the solution was done for 30min at 40°C. GC-MS analysis was done using Thermo Trace GC Ultra coupled with Thermo Fisher DSQ П mass spectrometers, with the chromatographic separation of metabolites using thermo TR50 column. The GC-MS analyzed data was processed using software Xcalibur. Xcalibur software was used to process the GC-MS analyzed data. The oven temperature was maintained at 70°C for 5 min, with its gradual increase at the rate of 5°C/min, 70°C to 310°C and stabilized for 5 min. The derivatized sample was injected in the split mode (splitting ratio of 1:16). The flow rate of carrier gas (Helium) was 1 mL/min. The MS detector was run in the electron impact (EI) mode, with electron energy of 70 eV. WILLY and NIST mass spectral library was used as reference data for the analysis of GC-MS processed results (Gupta et al., 2018). 

### Enhanced Biotic Stress Amelioration by *B. amyloliquefaciens* in Presence of Insoluble Si Source-Feldspar

*B. amyloliquefaciens* NBRISN13 (Nautiyal et al., 2013a), proven to be a good biocontrol agent against RS (Srivastava et al., 2016), was used as model strain to elucidate the role of silicon fertilization for improved biotic stress amelioration in rice under greenhouse conditions. Feldspar (Fe), the insoluble source of silicon, was used in the study @ 150 mg/Kg (Ranganathan et al., 2006). In brief, the 15-day old seedlings of the rice variety Jayanti (low Si accumulator and disease susceptible; **Supplementary Figure S6**) were transplanted to earthen pots (three seedlings per hill; three hills per pot) and after 3^rd^ day of seedling transplantation NBRISN13 was inoculated by soil drenching to maintain 20% moisture with bacterial culture (~1x10^7^CFU/ml). Biotic stress was given to the plants after one month of transplantation using the sclerotium of RS. The sclerotia sheathed in moist cotton were placed at the lowest sheath of tillers and the infected plants were covered with moistened polythene bags for 48h to sustain humidity in order to create favorable climatic conditions for growth and spread of pathogen (Srivastava et al., 2016). Experiment was performed in six replicates in earthen pots under greenhouse conditions with treatment details i) control (C), (ii) RS (RS), (iii) *B. amyloliquefaciens* (SN), (iv) *B. amyloliquefaciens +* RS (SN *+* RS), (v) Feldspar control (FeC), (vi) RS *+* Fe, (vii) SN *+* Fe and (viii) SN *+* RS *+* Fe. The sandy loam soil of CSIR- National Botanical Research Institute garden (latitude/longitude 11°24′N/79°44′E) was used for the greenhouse experiment with the following physio-chemical properties: pH-7.4 ± 0.2, EC- 125.67 µS/cm, TOC- 0.633%, total K- 2.34 mg/g, total N- 0.132409%, total P- 53.84076 µg/g, Si- 38.30 mg/kg. Disease severity as the measure of disease spread over the whole plant was quantified as number, length and diameter of lesions, and their distance from the girth after 15 days post infection (dpi); was also evaluated according to the rating scale as mentioned by Park et al. (2008) and physical parameters were measured after final harvesting of plants.

### Quantification of Si Uptake in Plant

Silicon uptake in leaf blade tissues of rice was performed through microwave digestion of oven dried plant tissue (0.1g) with 70% nitric acid and 30% hydrogen peroxide in duplicates. Digested mixture was subjected to overnight incubation at room temperature (RT), followed by addition of 5 ml of 10% sodium hydroxide, digested for 30 s to 1 min and kept for overnight in shaking condition. Quantitative estimation of Si uptake was performed by modified molybdenum blue method in triplicate (Nayar et al., 1975). In brief, digested sample supernatant was mixed with 2 volumes of ammonium molybdate (10%) and incubated at room temperature for 5 min, followed by addition of 2 volumes of 0.5% ascorbic acid and equal volume of 10% oxalic acid for removal of phosphorous interference followed by 5 volumes of 1:1 dil. HCl. The reaction volume was made up to 3 ml followed by 15 min incubation at RT. Absorbance was taken at 600 nm against water blank.

### Estimation of Chlorophyll, Proline, and Sugar Content in Shoot Tissues

Total sugar, chlorophyll, and proline content were estimated as mentioned by Srivastava et al. (2016). These estimations were performed in triplicate using fresh shoot tissues of rice at 7^th^, 15^th^, 30^th^, and 45^th^ day post infection (dpi) of RS.

### Determination of Antioxidant and Cell Wall Degrading Enzyme Activities

Antioxidant enzyme assays *viz*. superoxide dismutase (SOD), ascorbate peroxidase (APX), Glutathione peroxidase (GPX) and catalase (CAT) were determined using the method of Beauchamp and Fridovich (1971); Nakano and Asada (1981); Hemeda and Klein (1990) and Aebi (1984) respectively.

For estimation of cell wall degrading enzyme assays, the cryopreserved shoot tissues were homogenized in sodium phosphate buffer (pH 7.0) (20 mM cysteine HCl, 20 mM EDTA, and 0.5% triton X-100) and different cell wall degrading enzymes (CWDE) like polygalacturonase (PG), endopectate lyase (PL), β-glucosidase (BG), β-1,3 glucanase (Glu), and cellulase in the homogenate were measured as described earlier (Miller, 1959; Nautiyal et al., 2013a; Nautiyal et al., 2013b).

### Real-Time PCR Analysis

Total RNA was isolated from frozen leaf tissues of each treatment using the RNeasy Plant Mini Kit (Qiagen, Hilden, Germany) and was further used for first strand cDNA synthesis (maxima H minus cDNA synthesis kit, Fermentas Thermo scientific) as per manufacturer's instructions. Real-time PCR for randomly selected genes (**Supplementary Table S7**) using actin as an internal reference gene was carried out in triplicates, at the cycle conditions of an initial denaturation at 94°C for 5 min, followed by 35 cycles of 94°C for 30s, 60°C for 30s, and 72°C for 30s using Quanti Tect™ SYBR^®^ Green PCR kit (Qiagen) on stratagene Mx3000P systems. ct values obtained for each reaction was used to calculate the fold change using delta-delta ct method.

### Statistical Analysis

Wherever not mentioned, all *in vitro* experiments were performed in triplicate consecutively three times and results were subjected to statistical analysis by one-way ANOVA using SPSS 16.0 software. Results were represented as mean values of three replicates with significant statistical differences at p < 0.05 using Duncan test. Principal component analysis (PCA) was performed using XLstat (by Addinsoft; version 2019.4.1) statistical software.

## Results

### Role of Media Components in Designing P Discriminating NBRISSM Media

In a process of developing a defined medium for screening of Si-solubilizing microbes, different sources of Si have been amended in the medium. Among the Si sources (talc, magnesium trisilicate, and feldspar) used, magnesium trisilicate was chosen as the best Si source with a soluble Si in a range of 26.63 to 37.50 µg/ml (**Supplementary Table S1**). Addition of other Si sources *viz*. talc and feldspar; results in color change of the media (uninoculated) after autoclaving, therefore not suitable for qualitative screening of Si-solubilizing microbes (**Supplementary Figure S1**). Among different P sources tested, hydroxyapatite has been chosen as the most appropriate source having Si estimation ranging from 0.98 to 10.40 µg/ml as compared to KH_2_PO_4_ control (32.50–48.43 µg/ml) (**Supplementary Table S1**). Thus, the medium with magnesium trisilicate as silicon source and hydroxyapatite as phosphate source was chosen for further experimentation. The effect of different concentrations of hydroxyapatite (0.25%, 0.50% and 1.00%) was checked for Si and P estimations and it was marked that Si estimation was almost comparable in 0.25% and 0.50% as compared to 1.00%, therefore, lower concentration with less P quantification (0.00–4.4.55 µg/ml) was selected (**Supplementary Table S1**). In order to develop efficient screening medium different carbon (C) sources, as a source of energy and metabolism, were tested by replacing the glucose. Among them glucose itself and L-arabinose contribute to higher Si solubilization, however, L- arabinose was omitted due to its higher P-estimation values (6.50–33.80 µg/ml) than glucose (1.95–17.23 µg/ml). Lowest concentration of glucose (0.25%) was chosen for further experimentation because of minor differences in the amount of Si solubilized (180.00–323.63 µg/ml) by different concentrations of glucose (0.25%, 0.5%, 1% and 1.5%; **Supplementary Table S2**). Role of different nitrogen sources were then tested for choosing the appropriate N-source for Si solubilization. Among the six different nitrogen sources used, ammonium sulfate and ammonium chloride were selected for better Si solubilization. Evaluation of the appropriateness of the concentration showed that ammonium sulfate (0.01%) with the same concentration as in NBRIP medium gave almost similar results of Si solubilization (191.25–292.88 µg/ml) as in higher concentration (**Supplementary Table S2**).

**Figure 1:**
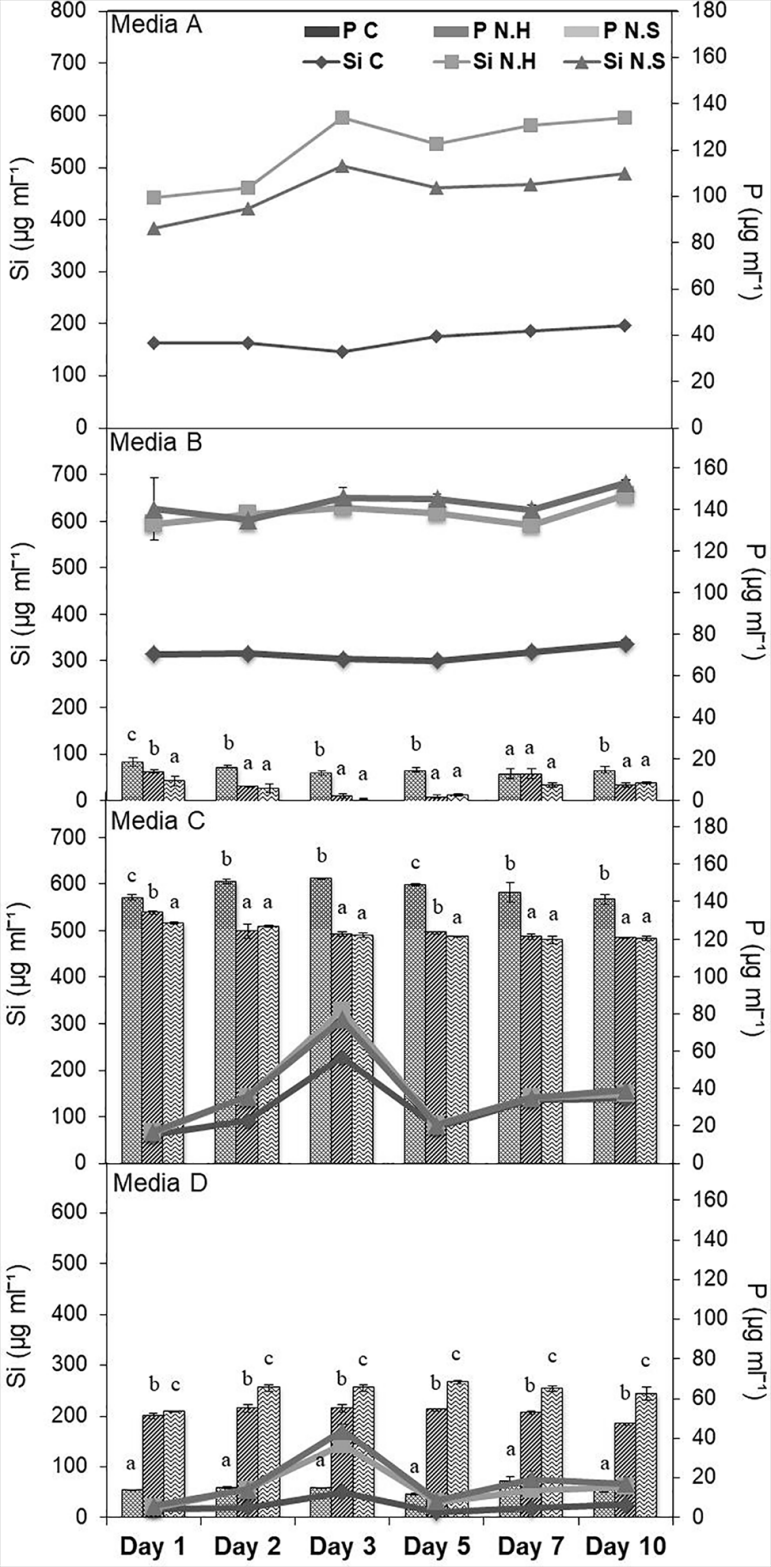
Comparison of NBRISSM (medium A), for its P-discriminating Si-solubilizing attribute with earlier reported media: B (Vasanthi et al., 2018), C (Sheng et al., 2008), and D (Nautiyal, 1999). Data are the average of three independent experiments and vertical bars indicate mean ± S.D. of three replicates.

Different salt sources were used to increase the efficiency of microbes to solubilize Si and it was found that estimated Si solubilization was higher in medium with calcium chloride and magnesium nitrate sources. Among different percentages of MgNO_3_ and CaCl_2_, the solubilization was comparable in all the three tested concentrations. Therefore, lowest concentration (0.125%) of both was selected with solubilized Si range 495.38–741.38 µg/ml, for the two bacterial strains chosen for the study (**Supplementary Table S3**). The media composition of NBRISSM (gl^−1^) thus finalized was: glucose (2.5); hydroxyapatite (2.5); MgNO_3_ (1.25); CaCl_2_ (1.25); (NH_4_)_2_SO_4_ (0.1); Mg_2_O_8_Si_3_ (0.1). During the comparative study among different reported Si-solubilizing media with our media showed that estimation of inorganic P was in decreasing order of medium C (119.60–152.43 µg/ml), medium D (11.70–68.25 µg/ml), medium B (0.98–18.53 µg/ml), and medium A (NBRISSM; 0.00 µg/ml) with least P estimations (**Figure 1**).

### Qualitative Media for Rapid Screening

After standardization of the quantitative medium, the pH indicator dye (BCP) was used to make the screening process more efficient (qualitative). The qualitative screening at 430 nm (absorption maxima of BCP) and quantitative estimation at 650 nm showed an inverse relation at 0.0025% dye concentration, because of bacterial inoculation. Inverse relation due to the shift in absorption maxima correlated well with the change in blue color and higher quantification was probably due to less hindrance of the dye (**Supplementary Figure S2**).

### Discrimination Between P and Si Solubilizers

**Figure 2:**
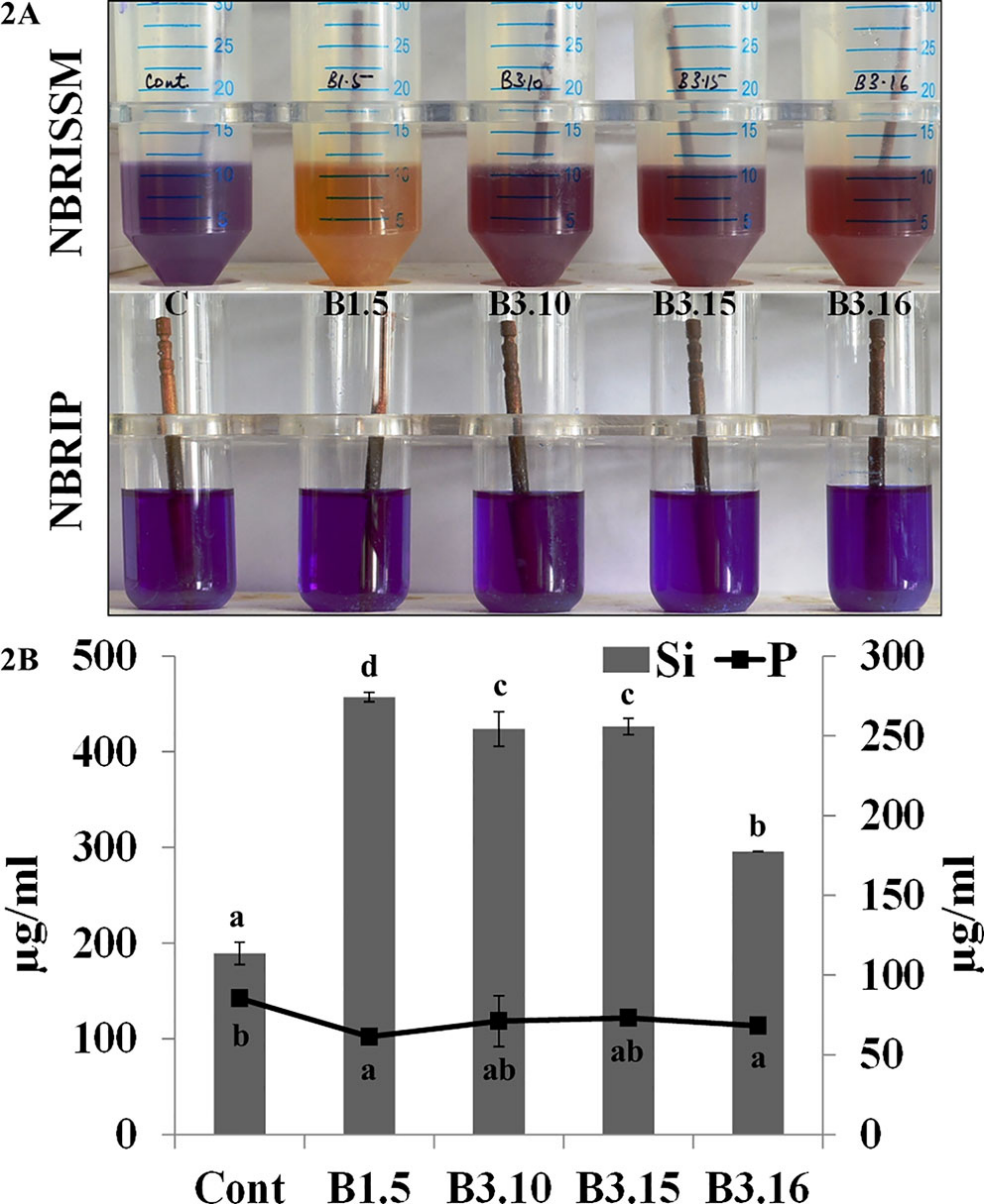
**(A)** Qualitative and **(B)** quantitative estimations of Si and P in NBRISSM and NBRIP medium. Results are the average of three independent experiments and vertical bars indicate mean ± S.D. of three replicates. Means followed by the same letter were not significantly different at p < 0.05.

Bacterial strains from different locations and rhizosphere were screened for Si solubilization using the designed medium. The solubilization was confirmed by the change in color of medium from purple toward yellow after the incubation at 28°C for 72h, however, some strains showed early color change and regain the blue color on further incubation. The strains which were not able to mineralize P, did not show early color change (blue to yellow on 1^st^ day), rather the color change observed on 3^rd^ day, which persisted until 5^th^ and 7^th^ day denotes Si solubilizer. Therefore, to avoid P mineralizers, early color changers were eliminated. Comparative quantification of Si and P in the NBRISSM medium after 5^th^ day of bacterial inoculation showed higher amount of solubilized Si as compared to phosphate. Absence of color change in NBRIP medium by the good Si solubilizer (B1.5, B3.10, B3.15, and B3.16) shows the noteworthy response of medium for screening Si solubilizers (**Figures 2A, B**).

The best Si solubilizers (vs9, vs14, and vs7) were found to solubilize P in NBRIP media containing TCP as inorganic source of P in the range 29.57–87.42 µg/ml, however, no solubilization of hydroxyapatite was observed by these strains in NBRISSM media. This shows that NBRISSM media give the quantification of only Si and there is no P interference, thereby clearly discriminating Si solubilizers (**Supplementary Figure S3**).

### Elucidation of Si Solubilization Mechanism

Microbes qualitatively screened for low, moderate, and high Si solubilizers were quantitatively compared for their Si and P solubilization, acid and alkaline phosphatase activity, alteration in pH, and organic acid production by growing them in NBRISSM medium (**Supplementary Figure S3**). Acidic phosphatase (Aci P) activity was highest in sp5 and vs9 followed by sp2 and sp4. On the other hand, alkaline phosphatase activity showed no remarkable difference among different Si solubilizers (**Supplementary Figure S3**). Lowering of pH was monitored in culture filtrate on 1^st^, 3^rd^, 5^th^, and 10^th^ dpi and remarkable change was found on 5^th^ dpi in NBRISSM media containing magnesium trisilicate as Si source and hydroxyapatite as P source. In accordance to the reports of Vasanthi et al. (2018), alteration in the pH by different strains was either lowered (acidic) in case of high Si solubilizers (5.42–6.75) or same as uninoculated media (7.41) but increased on 10^th^ dpi. Study on the role of organic acids during Si solubilization, the present study showed the production of maleic, succinic, and fumaric acid along with the known gluconic and tartaric acid during Si solubilization in media containing magnesium trisilicate as Si source. Among the five organic acids tested, maleic, succinic, and gluconic acids were the dominant acids, whereas tartaric and fumaric were produced only in vs4 followed by vs7 and sp1 (tartaric); vs7 and vs9 (fumaric) inoculated cultures (**Supplementary Figure S3**). PCA analysis (**Figure 5**) showed that Si solubilization, acidic phosphatase activity, tartaric acid, succinic acid, fumaric acid, maleic acid form close clusters, whereas gluconic acid and P solubilization in NBRIP media are distantly placed. Alkaline phosphatase activity was found to be distant from Si solubilization. F1 is the principal component showing the largest eigen value 16.992, proposing greater proportion of variability. F1 component explains 99.952% of the total variability contained in the original variables and shows positive correlation with all the variables. The other components explain gradually decreasing contributions (with eigenvalues <1). Variables gluconic acid and P solubilization in NBRIP media are the most isolated points on biplot graph. Hence, the production of maleic, succinic, and fumaric acid along with acidic phosphatase activity are the probable mechanisms of Si solubilization in NBRISSM media.

### Systematic Analysis of Silicon Solubilizers

**Figure 3:**
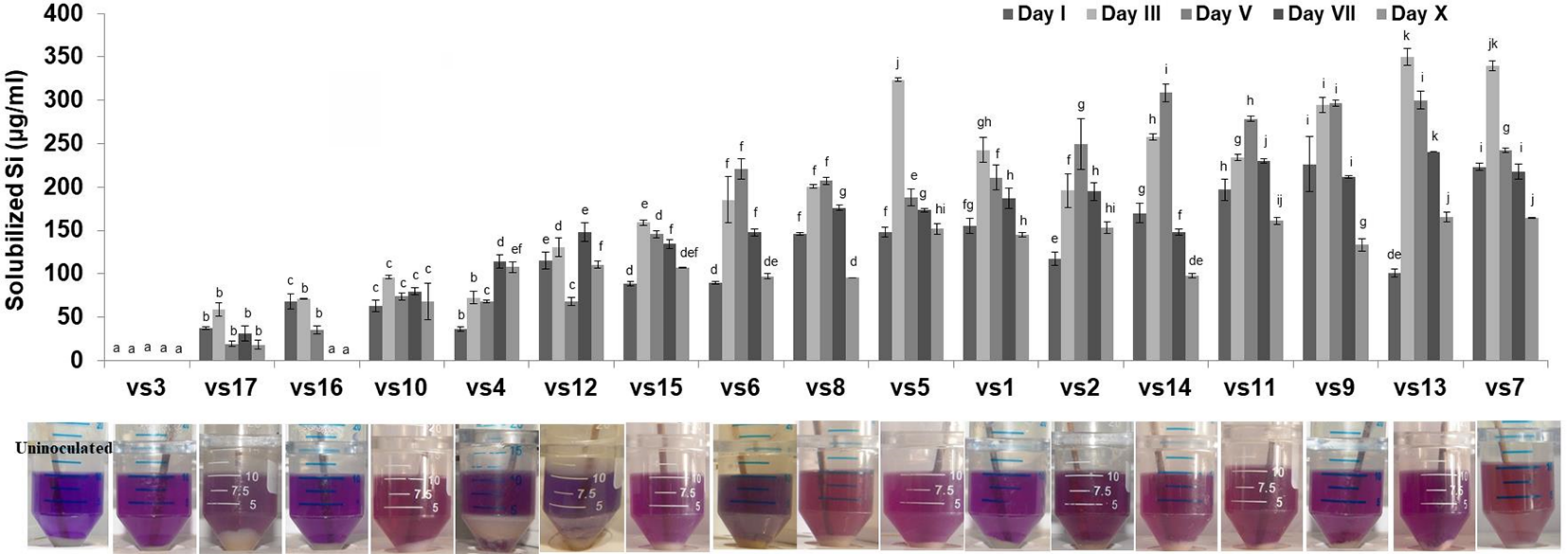
Quantitative estimation of solubilized Si among low, moderate, and high Si solubilizers at different time intervals, with their qualitative distinction as compared to the uninoculated control. Results are the means of three independent experiments. Vertical bars indicate mean ± S.D. of three replicates. Means followed by the same letter were not significantly different at p < 0.05.

**Figure 4:**
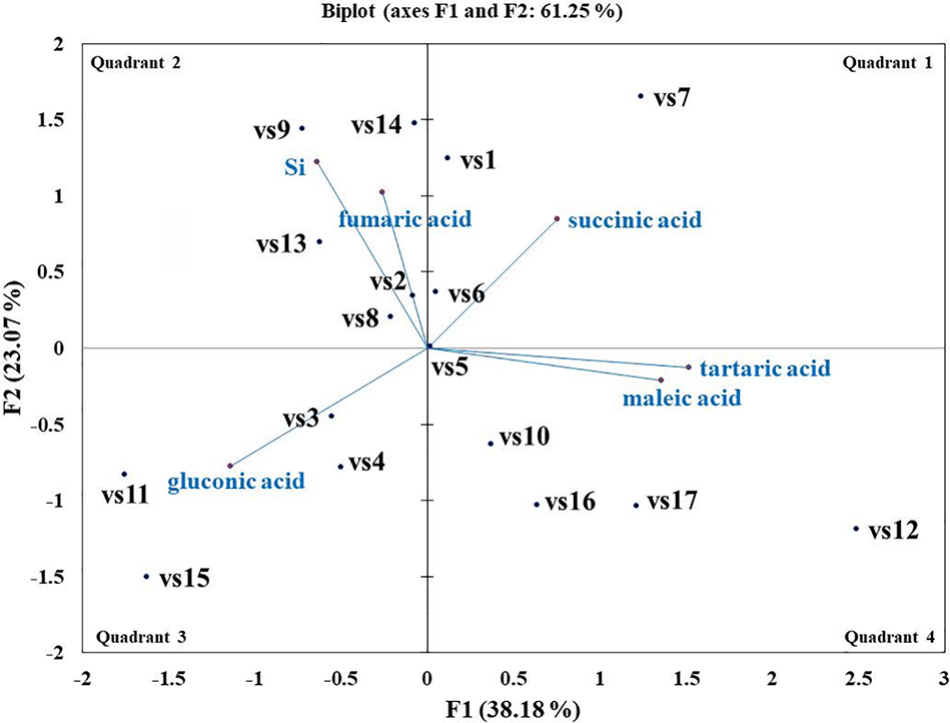
Biplot graph (with axes F1 and F2) of principal component analysis (PCA) to draw correlation among silicon (Si) solubilizers for Si solubilization and organic acid production mechanism.

Among the soil isolates, 17 strains with different level of Si solubilization were screened from 265 bacterial isolates of different locations (**Supplementary Table S4**). There were maximum number of Si solubilizers from Bulandshahr (Soil Si: 28–58.65 mg/kg) and few from Punjab (Soil Si: 15–17.25 mg/kg). Other isolates showed moderate or no Si solubilization even when the soil Si content was high. The selected strains were evaluated (both qualitative and quantitative) for their Si solubilization property up to 10 days of inoculation in NBRISSM. It was inferred that different strains showed distinct pattern of solubilization (high to moderate to low) (**Figure 3**). Systematic analysis using 16S rDNA based sequencing of the selected strains for silicon solubilization showed *Bacillus* and *Pseudomonas* as two dominant microbial genera. This analysis also suggests role of other *Pseudomonas* and *Bacillus* species in addition to *Sphingobacterium* sp. (NBRIVS7) to this list of predominant Si-solubilizing bacteria. However, in *Pseudomonas* phylogrouping major members belongs to *Pseudomonas alcaliphila* and *Pseudomonas* sp. with various different strains including *P. Chengduensis*, *P. nitroreducens*, and *P. veronii.* Species from *Bacillus* includes *B. safensis, B. subtilis*, *B. stratosphericus*, and *B. cereus* reported from present study. The phylogenetic relatedness can be observed from **Figure 6**. PCA analysis was performed, using statistical software XLstat (by Addinsoft; version 2019.4.1), to deduce the correlation between organic acid production and Si solubilization traits of Si solubilizers (**Figure 4**). The correlation biplot graph showed vs2 vs5, vs6, vs8 are closely clustered, and show least variation being closest to the origin, and also form same phylogenetic groups (**Figure 6**). Similar clustering is observed between vs1, vs9 and vs14; vs11 and vs15; vs3 and vs4; vs10, vs16, and vs17 belonging to either *Pseudomonas* or *Bacillus* genera. But vs3 and vs4; vs10, vs16, and vs17 form separate phylogenetic groups in the dendrogram but show similar function of silicon solubilization. Strains vs7 and vs12 are distinctly related. The three largest Eigen values are 2.291 (F1), 1.384 (F2), and 1.050 (F3) proposing greater proportion of variability by first three components. The first two components (F1 and F2) explain 38.176% and 23.071% of the total variability respectively accounting to 61.247% cumulative variance deciphering the greatest variance in the data and addition of a third component increases the total explained variability to 78.741%. The third component (F3) explains 17.494% of the variation, whereas, the remaining components present gradually decreasing contributions (with eigenvalues <1). The most isolated points in the biplot include vs7 and vs12 which plots separately (showing no cluster with others) in quadrants 1 and 4 respectively. Another correlation from **Figure 4** showed that Si solubilization clusters close to fumaric acid and is also close to succinic acid as compared to gluconic, maleic, and tartaric acids. Some new species of *Pseudomonas* and *Bacillus* along with a different strain of *Sphingobacterium* sp. have been identified in our present study.

**Figure 5:**
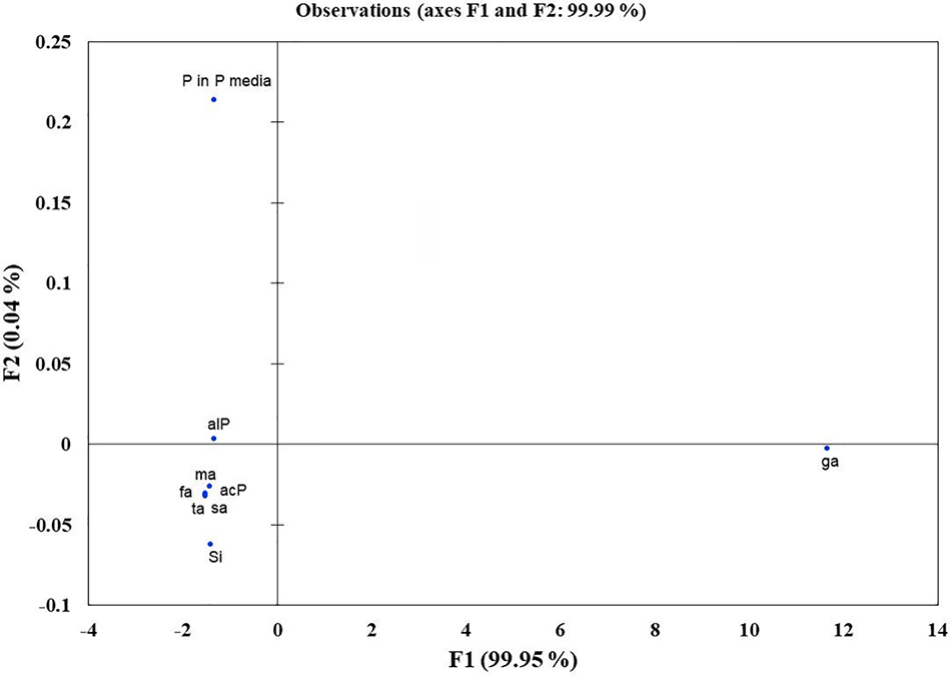
Principal component analysis to draw functional correlation among silicon solubilizers for different traits (variables). acP, acidic phosphatase activity; alP, alkaline phosphatase activity; Si, silicon solubilization; organic acids (ga, gluconic acid; ta, tartaric acid; ma, maleic acid; sa, succinic acid; fa, fumaric acid); P in P media, phosphate solubilization in NBRIP media.

**Figure 6:**
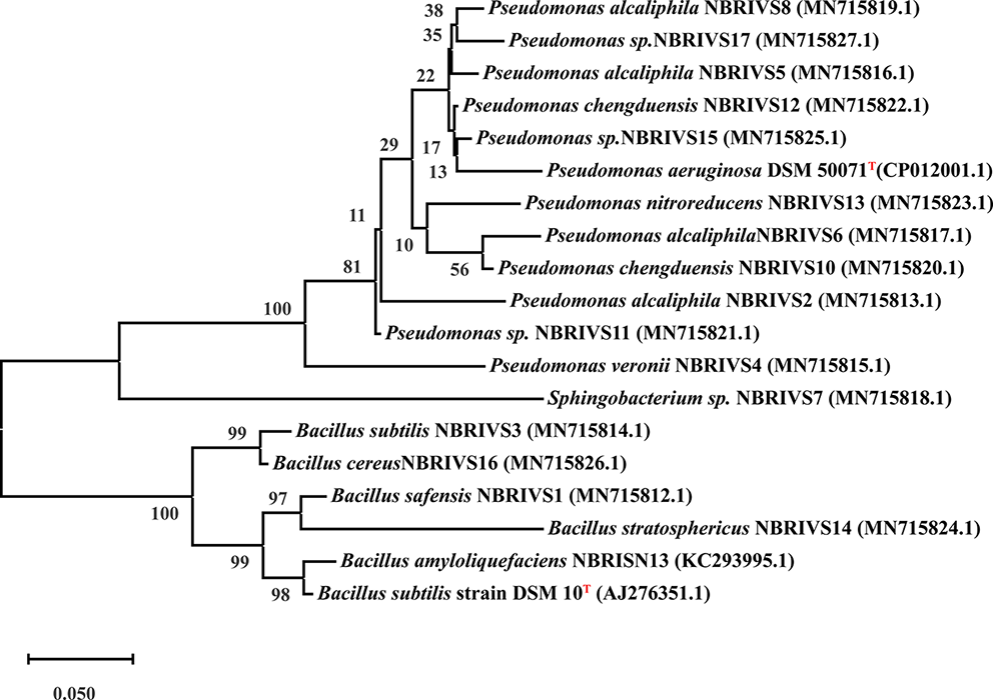
Phylogenetic analysis based on 16S rDNA (partial) sequences after MUSCLE alignment using MEGA10 software and applying Maximum Likelihood method (Kimura 2-parameter model) with 1,000 bootstrap replicates, considering complete sequence sites with type strains (T) marked as superscript.

### Metabolite Profiling during *In Vitro* interaction of NBRISN13-RS

NBRISN13 has already been reported to modulate plant metabolite system (Srivastava et al., 2016). Among the reported metabolites, six (succinic acid-M1, glycerol-M2, imidazole-M3, quinoline yellow-M4, quinazoline-M5, 2,3-dihydroxy benzoic acid-M6) were *in vitro* checked for their antifungal property by MIC (minimum inhibition concentration) assay. The results showed that metabolite M2 and M5 significantly reduced the growth of RS as compared to RS control. M2 showed 57.14% inhibition of fungal (RS) growth at 250 ppm concentration and 100% inhibition at 500 ppm whereas M5 showed 28.57% inhibition of RS at 250 ppm and 87.75% inhibition at 500 ppm (**Supplementary Figure S5**).

**Figure 7:**
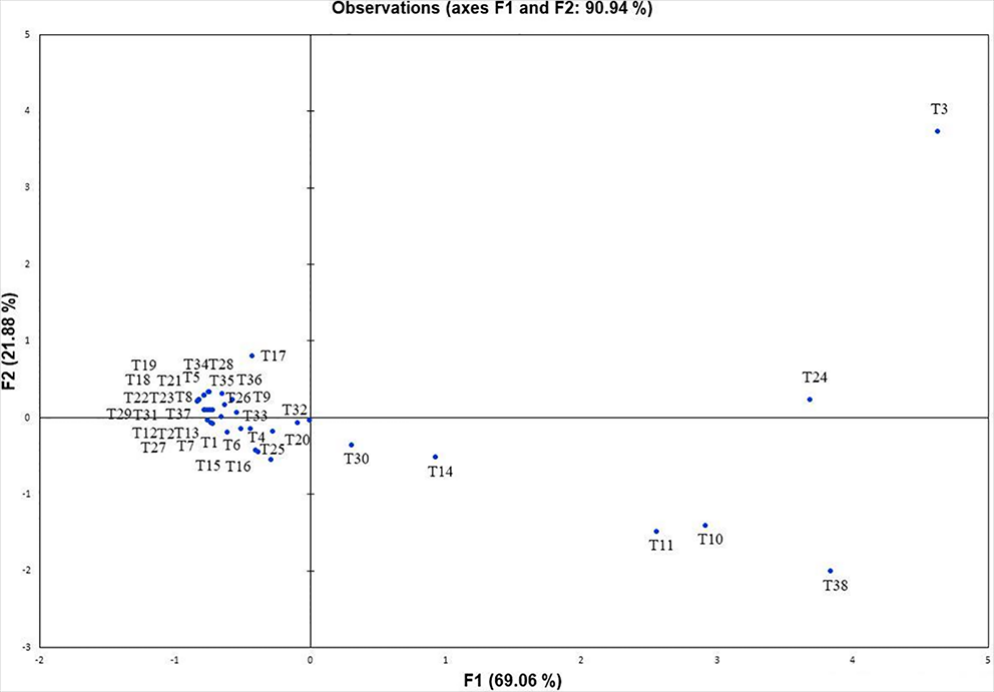
Principal component analysis to draw correlation among different metabolites identified during *in vitro* interaction between *Bacillus amyloliquefaciens* and *Rhizoctonia solani* (SN13-RS).

Further, to comprehend the role of NBRISN13 (vs9) in antagonism, its metabolic profiling was performed during *in vitro* interaction with RS through GC-MS profiling. Approximately 38 putative compounds were detected of which 21 are related to antimicrobial activity and defense signaling. The metabolites majorly identified in SN alone or during SN13-RS interaction include 5-Methoxymethyl-[1,3,4]thiadiazol-2-ylamine, Clarithromycin, 1, Dibutyl phthalate, Pyrrolo[1,2-a]pyrazine-1,4-dione, hexahydro-3-(2-methylpropyl)-, 13-Docosenamide, (Z)-, whereas Heptacosane, 2-Benzenedicarboxylic acid, bis(2-methylpropyl) ester, Octadecanoic acid, 2,3-bis[(trimethylsilyl)oxy]propyl ester, and Ergotaman-3′,6′,18-trione, 9,10-dihydro-12′-hydroxy-2′-methyl-5′-(phenylmethyl)-, (5′α,10α)- were principally identified in RS alone (**Supplementary Table S6**). Using PCA analysis, it was possible to draw correlation among the identified metabolites during the interaction between SN-RS. As shown in **Figure 7**, PCA elicits that the principal components PC1 and PC2 showed 69.06% and 21.88% variability respectively, of the total variation.

### Validation of NBRISN13 on Plant System as Enhancer of Biotic Stress Ameliorator

**Figure 8:**
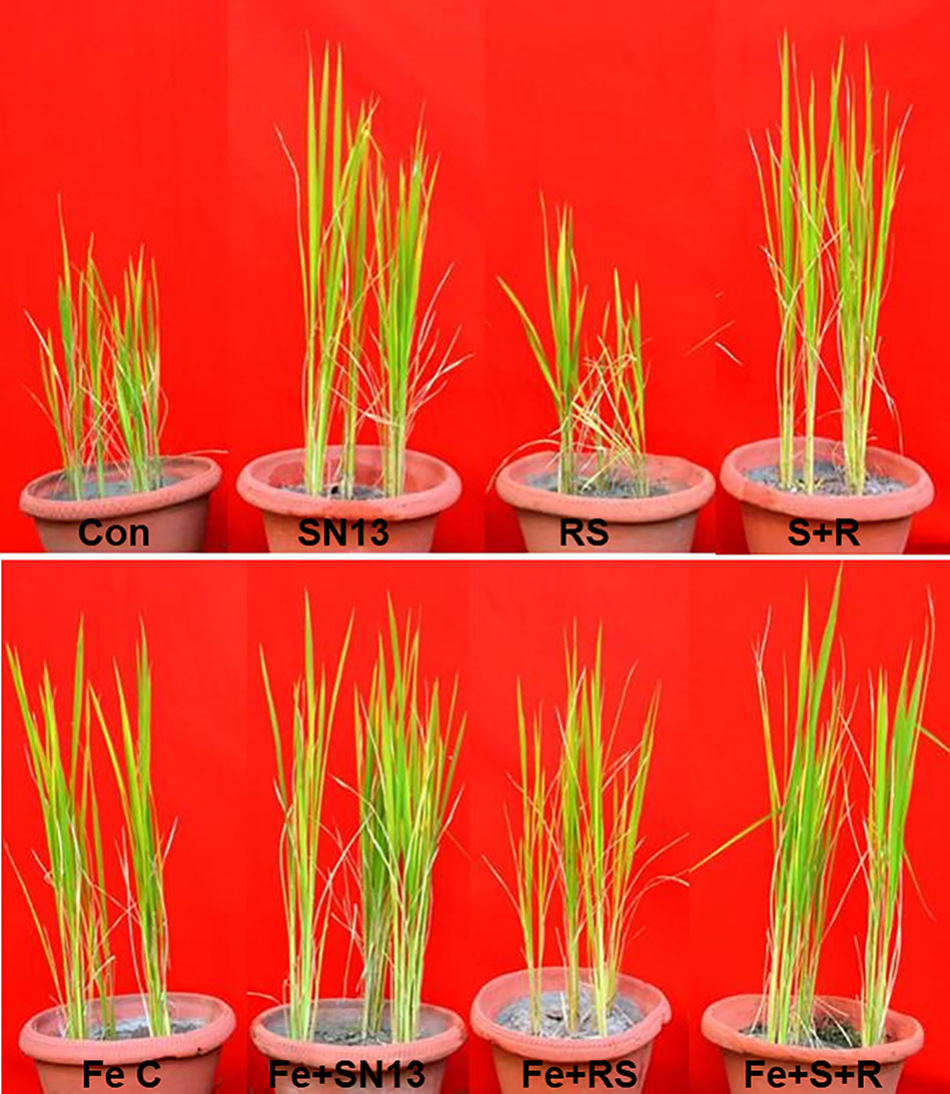
Interaction of Bacillus amyloliquefaciens (NBRISN13) and Rhizoctonia solani (R. solani) under control (CON, uninoculated control; SN13, NBRISN13 inoculated; RS, R. solani infected; S + R, SN13 + RS) and feldspar supplemented (Fe C, feldspar control; Fe + SN13, feldspar + NBRISN13; Fe + RS, feldspar + R. solani; Fe + S + R, feldspar + SN13 + RS) conditions during biotic stress of R. solani.Interaction of Bacillus amyloliquefaciens (NBRISN13) and Rhizoctonia solani (R. solani) under control (CON, uninoculated control; SN13, NBRISN13 inoculated; RS, R. solani infected; S + R, SN13 + RS) and feldspar supplemented (Fe C, feldspar control; Fe + SN13, feldspar + NBRISN13; Fe + RS, feldspar + R. solani; Fe + S + R, feldspar + SN13 + RS) conditions during biotic stress of *R*. solani.

Among screened Si solubilizers, six bacterial strains showed positive antifungal activity. *B. amyloliquefaciens*, NBRISN13 (vs9) being both a high Si solubilizer (133.50–226.12 µg/ml) and potent biocontrol agent (38.88%, **Supplementary Table S5**; Srivastava et al., 2016), was selected in present work for biotic stress amelioration study. It is clearly seen that infection of RS resulted (not a Si solubilizer; **Supplementary Figure S4**) in reduced shoot length (10.21%), number of spikes (18.18%), and dry weight (47.32%) as compared to control, however, was increased by SN13 in SN *+* RS. Increased shoot length was found in SN + Fe (13.95%) as compared to SN and SN *+* RS *+* Fe (4.70%) as compared to SN + RS. Number of spikes was increased by 10.00% in SN *+* RS *+* Fe as compared to feldspar deprived treatments. In presence of Fe dry weight showed the noteworthy results in RS *+* Fe (8.20%), SN *+* Fe (12.25%), and SN *+* RS *+* Fe (15.64%) as compared to RS, SN, and SN *+* RS respectively (**Table 1** and **Figure 8**).

**Table 1:** Effect of silicon (feldspar) supplementation and NBRISN13 on plant growth and disease severity of *Rhizoctonia solani* in rice under soil conditions.

Disease severity (15 dpi)								
	**Cont.**	**RS**	**SN**	**SN + RS**	**Fe C**	**RS + Fe**	**SN + Fe**	**SN + RS + Fe**
No. of spots	0.00 ± 0.00^a^	12.12 ± 1.11^e^	0.00 ± 0.00^a^	6.13 ± 0.29^c^	0.00 ± 0.00^a^	9.37 ± 0.49^d^	0.00 ± 0.00^a^	4.25 ± 0.31^b^
Length of spots (cm)	0.00 ± 0.00^a^	3.15 ± 0.47^c^	0.00 ± 0.00^a^	1.91 ± 0.18^b^	0.00 ± 0.00^a^	2.48 ± 0.42b^c^	0.00 ± 0.00^a^	1.87 ± 0.25^b^
Distance from the girth (cm)	0.00 ± 0.00^a^	15.45 ± 1.60^c^	0.00 ± 0.00^a^	8.05 ± 0.73^b^	0.00 ± 0.00^a^	14.07 ± 0.65^c^	0.00 ± 0.00^a^	7.47 ± 0.45^b^
Diameter of spots (cm)	0.00 ± 0.00^a^	0.85 ± 0.06^e^	0.00 ± 0.00^a^	0.40 ± 0.07^c^	0.00 ± 0.00^a^	0.63 ± 0.04^d^	0.00 ± 0.00^a^	0.18 ± 0.03^b^
**Physical parameters (45^th^ dpi)**								
Shoot length (cm)	51.88 ± 2.94^ab^	46.58 ± 5.04^a^	61.07 ± 2.62^bc^	57.97 ± 2.23^abc^	52.20 ± 3.21^ab^	49.15 ± 3.58^ab^	69.60 ± 4.06^c^	60.70 ± 6.43^bc^
Root Length (cm)	12.41 ± 0.75^bcd^	10.54 ± 0.87^ab^	13.41 ± 1.13^cd^	11.18 ± 0.36^bc^	10.56 ± 0.58^ab^	8.44 ± 0.67^a^	12.84 ± 0.61^bcd^	14.42 ± 1.17^d^
No. of spikes	1.37 ± 0.18^ab^	1.12 ± 0.29^a^	1.87 ± 0.23^bc^	1.25 ± 0.16^ab^	1.50 ± 0.19^ab^	1.25 ± 0.16^ab^	2.12 ± 0.23^c^	1.37 ± 0.18^ab^
No. of tillers	0.87 ± 0.23^ab^	0.75 ± 0.16^a^	1.5 ± 0.26^b^	1.13 ± 0.12^ab^	1.37 ± 0.18^ab^	0.87 ± 0.29^ab^	2.25 ± 0.16^c^	1.12 ± 0.12^ab^
Dry wt. (g)	4.86 ± 0.16^b^	2.56 ± 0.16^a^	6.77 ± 0.12^cd^	5.24 ± 0.56^b^	4.96 ± 0.39^b^	2.77 ± 0.24^a^	7.60 ± 0.61^d^	6.06 ± 0.38^bc^
**Biochemical characterization**								
Si content (mg/g DW) 7^th^	55.63 ± 3.13^b^	74.38 ± 8.13^c^	36.87 ± 0.63^a^	53.13 ± 3.13^b^	92.50 ± 6.25^d^	125.00 ± 1.25^f^	116.25 ± 5.00^e^	91.25 ± 3.75^d^
15^th^	161.87 ± 0.63^c^	61.25 ± 8.75^a^	153.75 ± 3.75^c^	104.37 ± 18.13^b^	187.50 ± 5.00^d^	166.87 ± 16.87^cd^	235.63 ± 5.63^e^	286.25 ± 27.50^f^
30^th^	158.75 ± 10.00^b^	77.50 ± 7.50^a^	178.75 ± 7.50^bc^	193.13 ± 6.88^c^	221.25 ± 8.75^d^	161.87 ± 11.87^b^	365.00 ± 12.50^f^	283.13 ± 20.63^e^
45th	164.37 ± 6.87^b^	98.75 ± 1.25^a^	211.87 ± 10.63^cd^	197.50 ± 7.50^c^	223.75 ± 1.25^d^	158.75 ± 3.75^b^	347.50 ± 20.00^f^	312.50 ± 2.50^e^
Total Chl (mg/g) 7th	0.12 ± 0.01^a^	0.14 ± 0.01^b^	0.17 ± 0.00^c^	0.14 ± 0.00^b^	0.21 ± 0.01^d^	0.14 ± 0.01^b^	0.21 ± 0.01^d^	0.16 ± 0.01^c^
15^th^	0.08 ± 0.00^a^	0.12 ± 0.00^b^	0.18 ± 0.01^e^	0.14 ± 0.01^c^	0.17 ± 0.00^d^	0.13 ± 0.01^b^	0.19 ± 0.00^f^	0.15 ± 0.00^c^
30^th^	0.06 ± 0.00^a^	0.09 ± 0.00^b^	0.15 ± 0.00^e^	0.13 ± 0.01^d^	0.13 ± 0.01^c^	0.09 ± 0.01^b^	0.17 ± 0.00^f^	0.14 ± 0.00^de^
45th	0.06 ± 0.00^a^	0.06 ± 0.00^a^	0.14 ± 0.00^e^	0.11 ± 0.00^c^	0.07 ± 0.00^b^	0.07 ± 0.01^a^	0.16 ± 0.00^f^	0.13 ± 0.00^d^
Proline (µM)7th	12.98 ± 0.22^ab^	20.73 ± 4.67^c^	9.79 ± 1.10^a^	16.61 ± 3.19^b^	33.71 ± 0.38^d^	12.65 ± 0.55^ab^	20.90 ± 2.75^c^	38.61 ± 0.33^e^
15^th^	10.34 ± 0.55^a^	14.41 ± 0.44^b^	13.64 ± 1.43^b^	20.68 ± 1.76^c^	28.98 ± 3.13^d^	9.46 ± 0.33^a^	14.96 ± 2.86^b^	16.33 ± 0.82^b^
30^th^	5.11 ± 0.35^a^	16.22 ± 0.13^e^	12.71 ± 0.57^c^	18.70 ± 0.44^f^	5.39 ± 0.11^a^	7.42 ± 0.03^b^	14.02 ± 0.41^d^	7.86 ± 0.03^b^
45th	3.46 ± 0.05^a^	15.84 ± 1.10^d^	6.87 ± 1.71^b^	11.77 ± 1.54^c^	2.53 ± 0.22^a^	7.48 ± 0.55^b^	2.86 ± 0.11^a^	3.19 ± 0.22^a^
Total sugar (µg/g)7_th_	53.10 ± 4.90^ab^	147.30 ± 10.70^d^	216.30 ± 0.70^e^	108.20 ± 8.40^c^	249.80 ± 10.40^f^	62.50 ± 6.90^b^	49.50 ± 4.70^ab^	40.90 ± 6.50^a^
15th	50.70 ± 3.30^a^	178.40 ± 8.00^e^	242.00 ± 14.00^f^	84.40 ± 11.40^b^	157.50 ± 6.50^d^	82.60 ± 1.40^b^	117.80 ± 9.60^c^	122.00 ± 5.40^de^
30^th^	56.30 ± 5.70^a^	214.90 ± 20.90^e^	180.90 ± 2.90^d^	108.00 ± 14.00^b^	106.30 ± 2.50^b^	141.00 ± 1.00^c^	134.70 ± 0.10^c^	307.40 ± 3.40^f^
45th	49.20 ± 4.60^a^	145.30 ± 7.10^d^	154.40 ± 9.80^d^	121.50 ± 7.70^c^	91.90 ± 1.30^b^	124.90 ± 0.90^c^	121.30 ± 1.50^c^	195.10 ± 0.90^e^

Mean values represented by different letters showed significant statistical differences at p < 0.05 using Duncan test.

Cont. indicates control; SN, *Bacillus amyloliquefaciens* (NBRISN13); RS, *Rhizoctonia solani*; Fe, feldspar.

The disease severity (**Table 1**) in various rice treatments were measured in terms of lesion/spot number, diameter, and length and their distance from the girth. The spot number, diameter, and their distance from girth was calculated by taking the average of each parameter of eight plants from six different biological replicates. The lesions observed were of necrotrophic type and more in RS and RS *+* Fe treatment. NBRISN13 being a biocontrol agent and Si solubilizer caused the reduction in disease. Feldspar treatment reduced the number of spots in RS *+* Fe by 22.68% which was further reduced to 64.94% by NBRISN13 in SN *+* RS *+* Fe treatment, as compared to RS resulting in reduced disease severity. Reduced spot length by Fe addition was found in RS *+* Fe (21.03%) and SN *+* RS *+* Fe (1.96%) as compared to RS and SN *+* RS respectively. Disease spread in terms of spot distance from the girth reduced in RS *+* Fe (8.89%) and SN *+* RS *+* Fe (7.14%) treatments as compared to RS and SN *+* RS. The diameter of spots also markedly showed the reduction in feldspar treatments (RS *+* Fe and SN *+* RS *+* Fe) as compared to RS and SN *+* RS (**Table 1**; **Supplementary Figure S7**).

Chlorophyll, sugar, and proline were assayed to study the biochemical and physiological status of plants. Reduced chlorophyll content in RS was rectified by SN13 in SN *+* RS significantly at 30^th^ (53.13%) and 45^th^ (74.64%) dpi comparative to 7^th^ (3.77%) and 15^th^ (18.58%) dpi. Feldspar addition improved the chlorophyll content in RS *+* Fe by 3% to 4.7%; SN *+* Fe by 7% to 22% and SN *+* RS *+* Fe by 2% to 15.7% as compared to RS, SN, and SN *+* RS treatments respectively. Increased sugar content in RS was lowered in SN *+* RS (16%–52.7%) by SN13 (7–45^th^ dpi). Fe addition initially increased the sugar in FeC which got lowered at 30^th^ and 45^th^ dpi. Constant lowering of sugar accumulation was noticed in RS *+* Fe (14%–57%) when compared to RS. Combined effect of SN13 and Fe was found to increase sugar content at later stage of infection (15–45^th^ dpi) in SN *+* RS *+* Fe as compared to SN *+* RS. Proline content in RS was found to be regulated by SN13 and feldspar at different stages of RS infection. SN *+* RS treatment showed increased accumulation at 15^th^ (43.51%) and 30^th^ (15.25%) dpi in comparison to RS. Feldspar supply showed high proline content in FeC at 7^th^ and 15^th^ dpi. Lowered proline accumulation was found in RS *+* Fe when compared to RS with further decrease in SN *+* RS *+* Fe (except at 15dpi).

Si uptake in rice leaves after 7^th^, 15^th^, 30^th^, and 45^th^ day post infection (dpi) of RS showed intensified Si uptake by SN13 in SN *+* Fe treatment as compared to FeC by 25.67% to 64.97% (7–45^th^ dpi). During infection, enhanced Si uptake occurred in SN *+* RS *+* Fe treatment by 71% to 96% as compared to RS *+* Fe at 15–45^th^ dpi, however, it was low at 7^th^ dpi.

**Figure 9:**
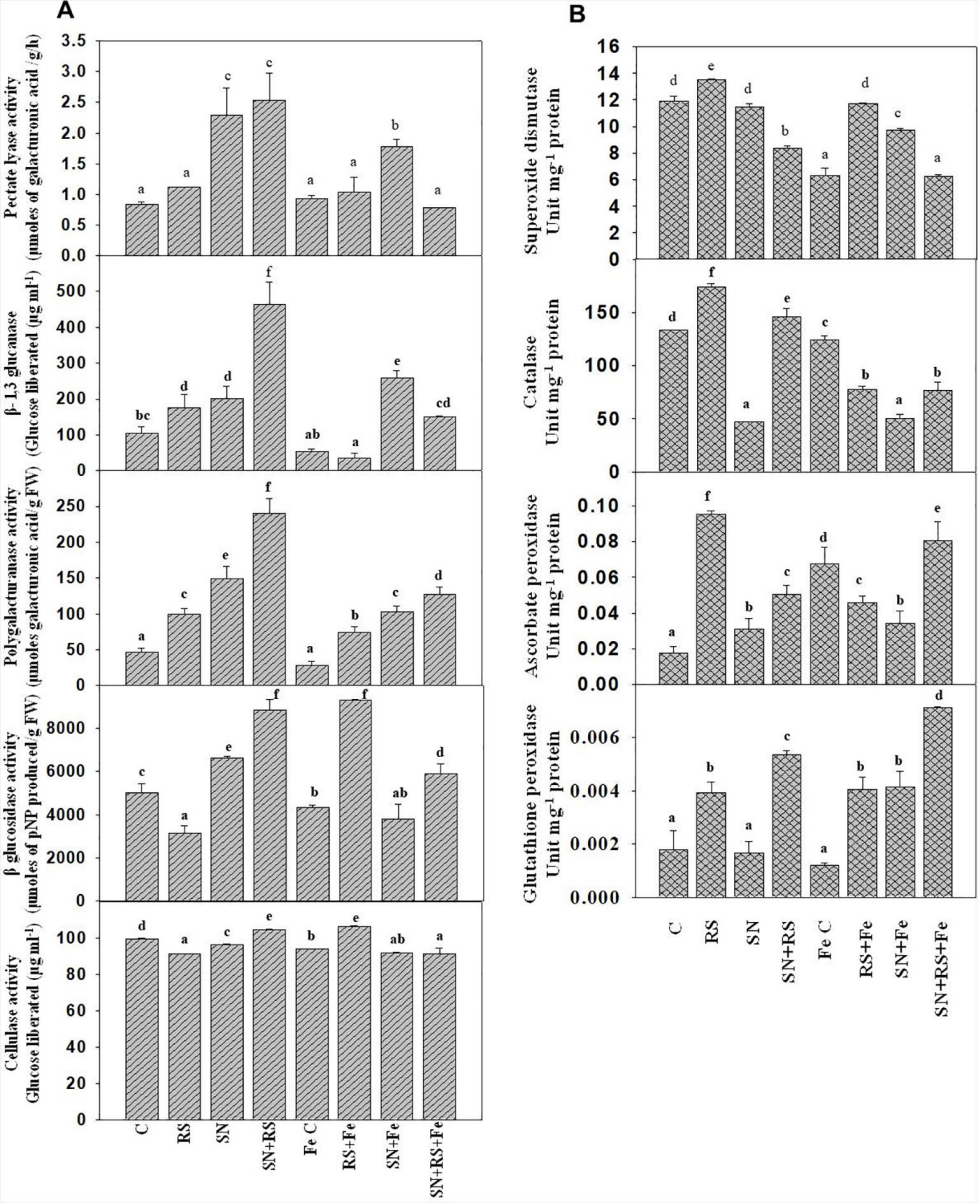
Synergistic effect of Feldspar supplementation and *Bacillus amyloliquefaciens* (NBRISN13) inoculation on cell wall degrading enzyme **(A)** and antioxidant enzyme **(B)** activities at 15 day post infection (dpi) of *Rhizoctonia solani* in rice shoot. Vertical bars indicate mean ± S.D. of three replicates. Means followed by the same letter were not significantly different at p < 0.05.

Cell wall degrading enzymes (CWDE) were estimated to evaluate their role during plant-pathogen interaction. Polygalacturonase (PG) activity was higher in RS (113.46%), SN (221.15%), and SN *+* RS (417.29%) when compared to control treatment. But the addition of feldspar (Fe) decreased the activity in aforementioned treatments and even in the FeC treatment. Fe lowered the activity in RS *+* Fe by 25.22%, SN *+* Fe by 30.54% and SN *+* RS *+* Fe by 46.84% when compared to RS, SN, and SN *+* RS respectively. **Figure 9A** showed increased production of Pectate lyase (PL) in SN (172.62%) and SN *+* RS (202.38%) treatments in comparison to control. Fe supplementation, however, reduced PL activity in SN *+* RS *+* Fe by 68.89% in comparison to SN *+* RS. Also the activity got lowered in RS *+* Fe when compared to RS but to lesser extent (by 7.14%). β-1,3 glucanase (Glu) was increased in RS (69.83%), SN (94.82%), and SN *+* RS (347.42%) when compared with uninoculated treatment. Addition of Fe reduced Glu activity in RS *+* Fe and SN *+* RS *+* Fe by 80.20% and 67.44% respectively in comparison to RS and SN *+* RS. In contrast to this, the activity was induced in SN *+* Fe treatment by 27.87% when compared with SN without Fe treatment. BG and cellulase both the activities were reduced in RS by 37.05% and 8.25% respectively whereas increased in SN *+* RS by 76.33% and 5.08% respectively. On addition of Fe, BG increased by 193.79% and cellulase by 16.81% in RS + Fe treatment; rather the activities were notably decreased in SN *+* Fe and SN *+* RS *+* Fe treatments (**Figure 9A**).

Antioxidant enzymes were assayed to study their role in modulation of oxidative stress. SOD, CAT, GPX, and APX activities were increased in RS alone treatment (13.26%, SOD; 30.69%, CAT; 1.29%, GPX; and 458.82%, APX) when compared with uninoculated control. Fe supplementation reduced the activities in RS *+* Fe (SOD by 13.41%, CAT by 55.39, APX by 52.63% and GPX by 2.56%). In SN *+* RS *+* Fe treatment, APX was higher by 77.77% and GPX by 75% when compared with RS *+* Fe treatment (**Figure 9B**). By modulation of different functions like Si uptake, cell wall degrading, and antioxidative enzymes, NBRISN13 reduce the disease severity and enhance the plant growth.

### Quantitative Real-Time PCR-Based Validation of Enhanced Stress Amelioration Due to Silicon Fertilization

**Figure 10:**
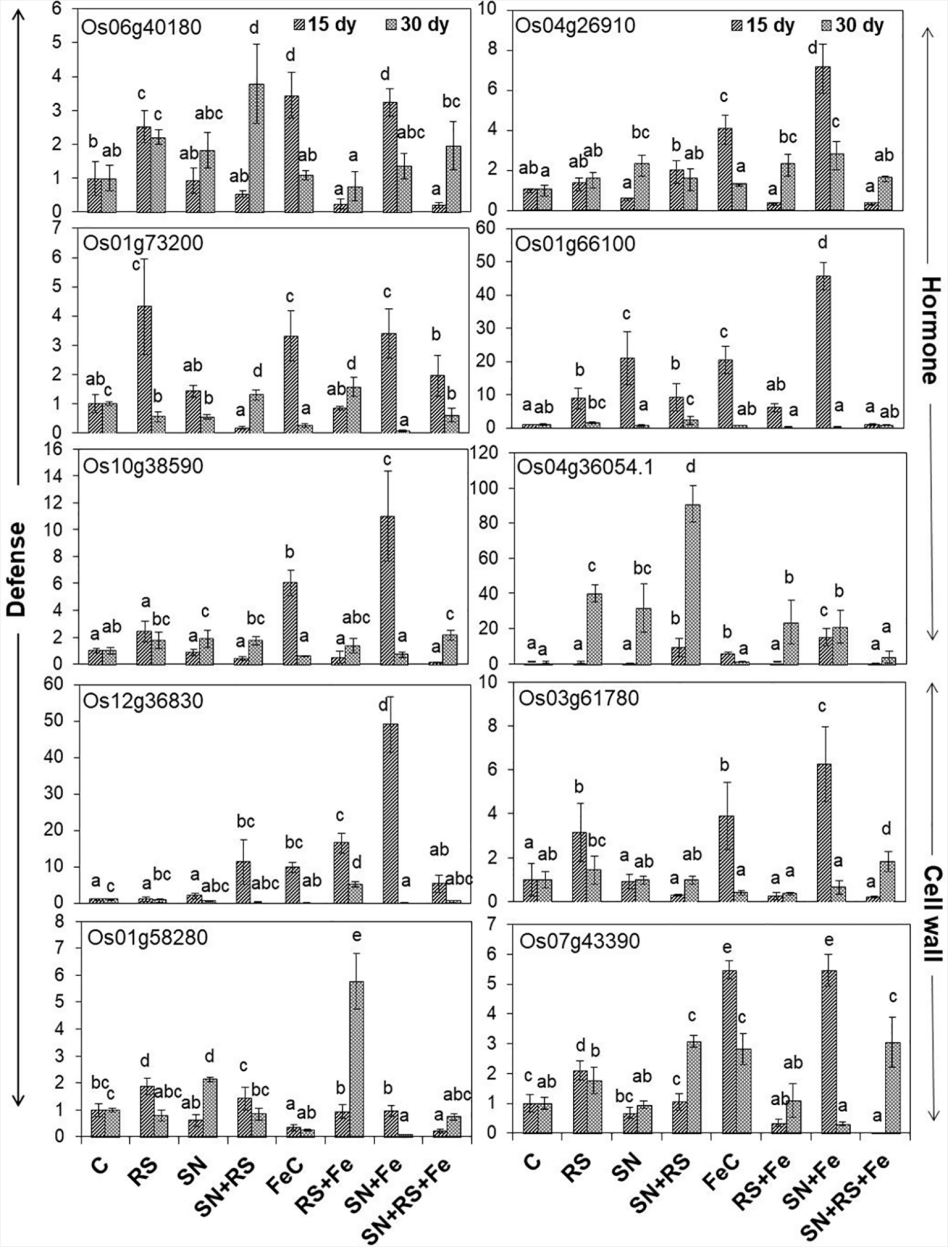
Relative expression of genes involved in defense, hormone, and cell wall modulations, through real time PCR analysis in shoot tissues of rice at 15th and 30th day post infection (dpi) of *Rhizoctonia solani*. Vertical bars indicate mean ± S.D. of three replicates. Means followed by the same letter were not significantly different at p < 0.05.

The present study also foster on the expression of genes (**Figure 10**) related to induced defense responses (**Supplementary Figure S7**) in plants at 15 and 30 day post infection (dpi) of RS. 4-α glucanotransferase (Os07g43390) having ~2 fold higher expression in RS at 15 dpi was lowered by 16.11% at 30 dpi (1.7 folds). Its expression was upregulated in SN *+* RS (three folds) treatment at 30 dpi. Addition of feldspar (Fe) lowered its expression in RS *+* Fe by 37% to 84% as compared to RS, both at 15 and 30 dpi. The expression was markedly higher in FeC (five folds) and SN *+* Fe (five folds) at 15 dpi which decreased at later stage of infection contrary to SN *+* RS *+* Fe at 30 dpi. Peroxidase precursor (Os01g73200) was increasingly upregulated in RS at 15 dpi (four folds) but significantly downregulated at 30 dpi. Upregulated expression due to feldspar supplementation in SN *+* Fe and SN *+* RS *+* Fe at 15 dpi, with significant downregulation at 30 dpi was also observed. Putative subtilisin homologue (Os01g58280) was found upregulated in RS at 15 dpi whereas, SN13 treatment either modulated or downregulated its expression in SN *+* RS, SN *+* Fe, and SN *+* RS *+* Fe both 15 at 30 dpi. The notable upregulation was found on Fe amendment in RS *+* Fe (5.76 folds). Pathogenesis-related Bet v 1 family protein (PR-10; Os12g36830) expressed in RS at 15 dpi was elicited on addition of Fe but downregulated at 30 dpi in RS *+* Fe. Feldspar resulted in induced defense by significant upregulated expression in SN *+* Fe (49 folds) at early stage of infection (15 dpi). Induced expression of auxin response factor 9 (Os04g36054) in RS (~40 fold) and SN *+* RS (~91 fold) treatments in comparison to control at 30 dpi; was downregulated in RS *+* Fe (23 fold) with least expression in SN *+* RS *+* Fe (~4 fold) at 30 dpi. Its expression was also found in SN *+* RS and SN *+* Fe at 15 dpi. Expression of oxidoreductase (Os04g26910) in RS (1.3- to 1.5-fold) was almost similar at 15 and 30 dpi which got upregulated on Fe addition in RS *+* Fe (2.2 fold) at 30 dpi. SN13 priming and Fe addition to plants induced its expression in SN *+* Fe at 15 dpi (seven folds). Increased expression of phospholipase D (Os06g40180) in RS (2.2- to 2.5-fold) was downregulated by the amendment of Fe in RS *+* Fe (0.2- to 0.75-fold) at 15 and 30 dpi. In SN treatments gene was induced at 30 dpi but showed downregulation on addition of Fe in SN *+* Fe and SN *+* RS *+* Fe. Fe supplementation also showed increased expression of Os06g40180 in FeC and SN *+* Fe at 15 dpi. Glucan endo 1,3 β-glucosidase (Os03g61780) expression higher in RS was downregulated by SN in SN *+* RS both at 15 and 30 dpi. Fe addition clearly showed its role in induction of plant resistance by its increased expression in FeC and SN *+* Fe treatments at 15 dpi. Fe supply also reduced its expression in RS *+* Fe in comparison to RS both at 15 and 30 dpi. The pathogen induced the upregulation of glutathione S-transferase (Os10g38590) in RS more at 15 dpi than 30 dpi but was lowered by Fe. But it showed upregulation in SN *+* RS *+* Fe (two folds). Upregulation of gibberellin 20 oxidase (Os01g66100) was especially found at 15 dpi except with least expression in SN *+* RS *+* Fe (~0.8- to 1.0-fold) both at 15 and 30 dpi. Significant lowering of its expression was found in RS *+* Fe in comparison to RS both at 15 and 30 dpi. These results conclude the elevation of defense response due to SN13 and Fe amendment priming the plants for elicited resistance against RS and reduced damage caused by pathogen by modulating expression of different genes.

## Discussion

Silicon, though available abundantly, remains unavailable to plants due to its poor solubility. Role of microbes in converting to its plant available form in order to enhance abiotic and biotic stress tolerance in plant is well established (Brindavathy et al., 2012; Santi and Mulyanto, 2018). However, in absence of high throughput media and method to screen silicon-solubilizing bacteria, there arises a need to develop a silicon-solubilizing media (NBRISSM) efficient to discriminate between Si from P. Among different Si sources screened, magnesium trisilicate chosen as best Si source was in accordance to the report of Vasanthi et al. (2018).

Sharing of same biochemical estimation method for Si and P (molybdenum blue method, Elliott and Synder, 1991; Fiske and Subbarow, 1925) and production of organic acid as a common mechanism led to estimate solubilized P as phosphate interference. To eliminate P interference, insoluble phosphate source (tri-Calcium phosphate, TCP) from NBRISi (**Supplementary Table S1**) media was replaced with other P sources ***viz***. sodium phytate and hydroxyapatite for minimum P interference, as compared to soluble P source, contrary to the report of Sheng et al. (2008) and Vasanthi et al. (2018). Production of organic acids is known for solubilization of inorganic P (TCP) and Si solubilization (Sheng et al., 2008), hence, in order to study Si solubilization other than P solubilization, use of hydroxyapatite would be better as its mineralization involves phosphatases rather than organic acid production (Khan et al., 2007). The present study also showed hydroxyapatite as a better P source leading to less interference by the soluble P probably due to the involvement of acid phosphatase.

Sharing of P solubilization mechanism with Si also restrict the identification of microbes with Si solubilization property. Comparison of the designed medium with other reported silicon media (Sheng et al., 2008; Kang et al., 2017; Vasanthi et al., 2018; Lee et al., 2019) showed that NBRISSM has the best reproducibility in terms of Si solubilization and least P interference. Designing of such specific medium for screening of silicon-solubilizing microbes would be salutary and an option of using the second most available element of earth's crust in agriculture through green technologies for sustainable development.

Most of aforementioned media for screening of Si-solubilizing microbes are either based on the plate assays for their zone formation ability (Kang et al., 2017; Lee et al., 2019) or liquid media containing soluble P sources like K_2_HPO_4_ and Na_2_HPO_4_ (Sheng et al., 2008; Vasanthi et al., 2018). There are no prior reports about the preparation of defined medium for high throughput screening (qualitative, indicator dye based) of silicon-solubilizing bacteria with discrimination of P solubilization. The present study has validated the various amendments in the medium for screening Si-solubilizing microbes based on their quantitation and finalizes the different components of NBRISSM media as stated earlier.

Si solubilization is an organic acid production based phenomenon resulting in the change in color of medium. The essential part of this acidification could be attributed to the consumption of glucose from the growth medium leading to the production of organic acids (Mardad et al., 2013). According to Kang et al. (2017) acidolysis is the only common mechanism known for dissolution of silicate minerals. So, to elucidate the same in the designed media pH dependent attributes, *viz.* organic acid production and acid/alkaline phosphatase activities were determined. Comparative data showed that strains may have one or more traits together. From our findings (**Figure 5**), acidic phosphatases associated with lowering of pH is directly proportional with high Si solubilizers indicating that different pH dependent events are directly related with Si solubilization whereas; alkaline phosphatase has no distinct role among different strains. In the earlier report of Vasanthi et al. (2018), lowering of pH was observed in media containing magnesium trisilicate; similar pH lowering was found in NBRISSM media as evident from the color change. Functional correlation (PCA; **Figure 5**) of Si solubilization with acidic phosphatase activity is reported for the first time in our study.

Lowering of pH due to the activation of acid phosphatases for mineralization of organic phosphates has been stated by Naureen et al. (2015). The screening of bacterial strains using designed media also showed early change in color probably due to the involvement of acid phosphatases. Therefore, to avoid P mineralizers, early color changers were eliminated.

Earlier Vasanthi et al. (2018), reported gluconic, tartaric, acetic, and hydroxypropionic acid production using quartz and feldspar as Si source. In present study, organic acid production in magnesium trisilicate supplemented NBRISSM media has been reported first time. Fumaric, succinic, and maleic acids are the new organic acids reported in the present study, however, production of gluconic and tartaric acids are in accordance to Vasanthi et al. (2018).

Systematics of the selected strains for Si solubilization in the present study showed different microbial genera especially *Bacillus* and *Pseudomonas*. Genus *Bacillus* and *Pseudomonas* both have been reported to contain Si-solubilizing attribute (Sheng et al., 2008). *Sphingobacterium* sp. (NBRIVS7) is also identified among the predominant Si solubilizers *Bacillus* and *Pseudomonas* spp., which is not yet reported earlier for this function. Our findings also report some new *Bacillus* and *Pseudomonas* species other than those reported earlier for silicon solubilization (Vasanthi et al., 2018; Chandrakala et al., 2019; Lee et al., 2019). PCA analysis yield insights into the correlation of different Si solubilizers with organic acid production, results from the biplot graph also suggests the principal role of organic acids, especially fumaric and succinic (**Figure 4**), in Si solubilization as reported earlier (Sheng et al., 2008; Vasanthi et al., 2018). Functional correlation based on eigen values among different variables indicates the close relatedness between acidic phosphatase activity and Si solubilization, which is not reported earlier (**Figure 5**). Our findings showed the close relatedness among various strains predicting their similarity of functional and genetic characters. From the study of PCA and 16s rDNA sequence based dendrogram it was found that irrespective of different genus, functional similarity was present among the Si solubilizers.

After the screening of efficient biocontrol agent, *B. amyloliquefaciens*, NBRISN13 (vs9) comes out to be the high Si solubilizer as well as strong antifungal agent effective against RS responsible for sheath blight disease in accordance to the earlier report (Srivastava et al., 2016). As per earlier report (Srivastava et al., 2016) different metabolites secreted in rice tissue during SN13-RS interaction, glycerol (57.14%–100%) and quinazoline (28.57%–87.75%) were found inhibitory against RS under *in vitro* condition (**Supplementary Figures S5A**, **B**). Their potent antagonistic property shall have future implications of developing bio-pesticide with plant based metabolites, after further validation. Further metabolic analysis under *in vitro* SN13-RS interaction showed production of different metabolites principally involved in antimicrobial activity *viz.*, 5-Methoxymethyl-[1,3,4]thiadiazol-2-ylamine, Clarithromycin, Dibutyl phthalate, Pyrrolo[1,2-a]pyrazine-1,4-dione, hexahydro-3-(2-methylpropyl)-, 13-Docosenamide, (Z)-, Heptacosane, 2-Benzenedicarboxylic acid, bis(2-methylpropyl) ester, Octadecanoic acid, 2,3-bis[(trimethylsilyl)oxy]propyl ester (**Supplementary Table S6**). This metabolic study thus opens future approaches for developing bio-pesticides after their efficacy assessment (revalidations).

*B. amyloliquefaciens* was further assessed for disease suppression/mitigation in presence of insoluble Si source- Feldspar (economic source). The growth of rice plants measured in terms of root-shoot length and dry weight clearly showed the remarkable effect of NBRISN13 and feldspar amendment by increasing the shoot length, number of spikes, and dry weight in all the treatments as compared to control and RS treated plants (**Figure 8**). The plant growth promoting ability of NBRISN13 was further intensified on feldspar amendment as reported earlier by Peera et al. (2016), where, they showed the growth and yield improvement with Si source and silicate solubilizing bacteria. Feldspar treatment has been reported to confer pathogen resistance by providing physical barrier to pathogen infection or the induction of defense (Shen et al., 2010). The positive effect of Fe in terms of better plant growth and lesser disease development has been reported earlier by Abed-Ashtiani et al. (2012). In accordance to this, present study also reports increased dry mass in presence of feldspar (**Table 1**), even in RS *+* Fe treatment. This shows that Fe has no interfering effect on infection mechanism used by RS as reported by Shen et al. (2010), rather presence of Si solubilizer help the plants through cell wall strengthening and improved nutritional status (Meena et al., 2014). Furthermore, presence of SN13 along with Fe had both increased plant growth and reduced RS infection.

Disease suppression was also correlated to microbe (NBRISN13) mediated Si uptake in plants (Brindavathy et al., 2012). Higher uptake in SN + Fe showed the significant role of SN13 in dissolution and uptake of silicon (feldspar). Increased Si uptake also correlates to increased plant growth and biomass (**Table 1**). Enhanced Si uptake mediated induced defense response in plants (Wang et al., 2017) correlated well with the regulation of defense responsive genes in present study (**Figure 10**). Contribution of silicon to disease resistance was remarkably seen in SN *+* RS + Fe treatment (**Table 1**), as evident by reduced disease severity. It therefore acts as the preventive measure and also plays role in delayed initiation of disease (**Supplementary Figure S7**). Number, length, and diameter of RS infection spots were significantly found to be reduced in SN + RS + Fe on addition of feldspar and SN. This also correlates to enhanced resistance imparted by induced antioxidative enzyme responses (Malhotra et al., 2016).

Physiological and nutritional status of plant was determined in terms of total chlorophyll, proline, and total sugar. Reduced chlorophyll content by RS infection was improved by SN13 (Srivastava et al., 2016). Improved chlorophyll content in SN *+* RS *+* Fe and RS *+* Fe as compared to RS justifies role of silicon in plant growth metabolism (Cooke and Leishman, 2016; Al-Garni et al., 2019). Different patterns of sugar and proline accumulation was found to be regulated by SN13/SN *+* Fe, thereby reducing RS induced biotic stress. Reduced proline content in SN *+* RS contributes to lower oxidative damage due to detoxification of ROS (Dar et al., 2016). Role of sugar accumulation was reported for regulation of PAMP triggered immunity in plants (Morkunas and Ratajczak, 2014). Increased sugar accumulation in SN *+* RS *+* Fe treatment predict the role of sugar as priming molecule for induced plant defense.

As per the report of Meena et al. (2014), modifications in terms of cell wall degrading enzymes (**Figure 9A**) after 15 days of pathogen infection in presence and absence of Fe supplementation was observed. Polygalacturonase (PG) activity got lowered in presence of feldspar which directly proportionate to reduced pathogenic effect of RS (Gawade et al., 2017). In our study, lowering of PG in the RS treatments after the addition of Fe also corresponds to reduced loosening of cell wall which otherwise occurs due to breakdown of homogalacturonan by PG (Babu and Bayer, 2014). Pectate lyase (PL) reported for the breakdown of polysaccharides in plant cell wall thereby inducing defense (Vorwerk et al., 2004). Higher PL activity of SN13 showed the defense priming in plants whereas Fe addition lowered the cell wall damage in RS *+* Fe compared to RS control and also in SN *+* RS *+* Fe. Cellulase and BG were higher in RS *+* Fe treatment which might be showing the modulation in sugar accumulation (Srivastava et al., 2016).

Si fertilization in plants, already reported to induce defense responses, boost their resistance against various biotic stresses (Alhousari and Greger, 2018). The defense-related enzyme activities APX and GPX were markedly increased in treatments with feldspar compared to the control treatments (SN, RS, SN *+* RS) especially in SN *+* RS *+* Fe; proposing induced defense due to combinatorial effect of Fe and SN13 potent in minimizing the oxidative stress. From the report of Medrano-Macías et al. (2018), Si perform twofold function to attenuate antioxidant metabolism- either by increased activities of enzymes or reduced ROS production. Likewise, our findings showed decrease in SOD and CAT (Debona et al., 2014) on Fe addition proposing the suppressed colonization of RS whereas APX and GPX were higher.

Molecular insights on gene expression related to induced defense responses suggests their role during plant-pathogen interaction for increased resistance. Higher expression of 4-α glucanotransferase in RS at 15 dpi and SN + RS at 30 dpi was lowered by feldspar amendment. Their role in cell wall expansion (cellular metabolism) and starch degradation (conversion to sucrose), stipulate the induction of defense at early stages (Mantri et al., 2010; Nautiyal et al., 2013a). Feldspar addition thus showed its role in decrease of these stress responses. Peroxidase precursor (Os01g73200) reported for scavenging hydrogen peroxide (H_2_O_2_) radicals (Silveira et al., 2015; Srivastava et al., 2016), showed upregulation in RS at 15 dpi indicating ROS production during early stage of pathogen infection (Schopfer et al., 2001). Role of SN13 and Fe in ROS detoxification can be predicted in SN + RS + Fe at 15 dpi. SN13 mediated reduced expression in SN + RS with further downregulation of subtilisin homologue (Os01g58280) due to Fe, correlates to reduce stress (Srivastava et al., 2016). The results also justify its role in pathogen recognition earlier reported by Figueiredo et al. (2014). Bet v 1 family protein (Os12g36830) reported to be induced during pathogen attack as PR-10 proteins, was lowered by feldspar in RS + Fe. Its popular role in prevention of pathogen invasion was found in SN + RS when compared to control at 15 dpi which was further lowered in SN + RS + Fe. Induced defense by its upregulation in SN + Fe at early stage of infection envisage the antifungal nature of PRs (Ebrahim et al., 2011; Srivastava et al., 2016). Downregulation of auxin response factor 9 (Os04g36054) in RS + Fe and SN + RS + Fe at 30 dpi due to feldspar supplementation propose induction of auxin mediated defense signaling, thus conferring resistance against necrotrophic pathogens (Wang and Fu, 2011). Oxidoreductases already reported for the detoxification of reactive aldehydes produced during oxidative stress, showed upregulation by feldspar addition. The reduced expression in SN + RS + Fe contrary to RS and RS + Fe exhibits the combined effect of SN and Fe in maintaining antioxidant defense mechanisms of plants (Sengupta et al., 2015). Phospholipase D (Os06g40180) known for its role in lipid metabolism and cellular processes (Zhao, 2015) was downregulated by feldspar in RS + Fe at 15 and 30 dpi treatments. Its downregulation in SN + RS + Fe propose Fe mediated regulation of plant defense responses against abiotic and biotic stresses. Downregulation of glucan endo 1,3 β-glucosidase (Os03g61780) by SN13 in SN + RS both at 15 and 30 dpi and reduced expression in RS + Fe in comparison to RS both at 15 and 30 dpi signify the role of silicon in minimizing fungal invasion by regulating plant resistance to pathogen attack (Ma and Yamaji, 2008; Shen et al., 2010). Glutathione S-transferase (Os10g38590) showed higher expression in SN + RS + Fe (30 dpi) treatment thereby inducing the defense. Its expression was found to be lowered in RS + Fe at 30 dpi treatment predicting its regulation during disease development in plants. This confers its role as the marker for defense responses (Thatcher et al., 2015) especially the plant antioxidative defense to reduce excess ROS induced damage (Gullner et al., 2018). Upregulation of gibberellin 20 oxidase 2 (Os01g66100) at 15 dpi indicates disease development in plants (Qin et al., 2013) which was minimally expressed in SN + RS + Fe both at early and later stage of infection.

## Conclusion

The present work proposes an efficient screening medium for silicon-solubilizing microbes which is more discriminative between Si and P solubilization, in comparison to the earlier reported medium. The P source used in the medium make it more appropriate for the identification of silicon-solubilizing microbes thereby eliminating phosphate interference. Our findings suggest functional correlation of Si solubilization with acidic phosphatase activity which is firstly reported in our work, and organic acid production (gluconic, succinic, fumaric, tartaric, and maleic acid). Functional diversity studies showed genetic relatedness of *Bacillus* and *Pseudomonas* sp. dominance for this function. NBRISN13, a Si solubilizer, reported as biocontrol agent in our previous study, contributed to reduced disease severity with Feldspar supplementation. The study also reveals the role of its metabolite in antagonism against RS. Thus, NBRISSM would be a pertinent medium competitive enough for screening of Si solubilizers with remarkable differentiation between P and Si estimations. Increased silicon uptake in plants by NBRISN13 could be further applicable against different biotic and abiotic stresses as biofertilizer. The elucidation of the role of silicon-solubilizing bacteria in disease suppression entails production of silicon-solubilizing microbe based biofertilizers with antagonistic properties for future field applications. This manuscript thus describes potentially interesting method for screening silicon-solubilizing microorganisms and the importance of microbe-mediated Si uptake for improved plant health without use of pesticides.

## Data Availability Statement

All datasets for this study are included in the article/**Supplementary Material**.

## Author Contributions

VB and SS designed the work in association with other authors. VB, SS, MR, AN and KS performed the experiments. VB, MR, SS, AN, and AL performed the data analysis.

## Conflict of Interest

The authors declare that the research was conducted in the absence of any commercial or financial relationships that could be construed as a potential conflict of interest.
