# Supplementary material for: Silicon-Solubilizing Media and Its Implication for Characterization of Bacteria to Mitigate Biotic Stress
Source: Front Plant Sci. 2020 Feb 28;11:28. doi: 10.3389/fpls.2020.00028 (PMC7061934; doi:10.3389/fpls.2020.00028)
Supplement: Supplementary file 1 [file DataSheet_1.docx]

**SUPPLEMENTAL MATERIAL**

**Silicon-Solubilizing Media and Its Implication for Characterization of Bacteria to Mitigate Biotic Stress**

[***Vidisha Bist***](https://www.frontiersin.org/people/u/344475) ***1,2,  Abhishek Niranjan1,***[***Manish Ranjan***](https://www.frontiersin.org/people/u/856424) ***1,  Alok Lehri1,  Karishma Seem1 and [Suchi Srivastava](https://www.frontiersin.org/people/u/304805) 1,2****

1 Division of Microbial Technology, CSIR-National Botanical Research Institute, Lucknow, India

2 Academy of Scientific and Innovative Research (AcSIR), Ghaziabad, India

Table S1 Effect of various silicon and phosphate sources on silicon and phosphate solubilization by bacterial strains [B1 (*Bacillus safensis*) and B2 (*Bacillus amyloliquefaciens*)] in NBRIP broth media

|  | Si solubilization |  | P solubilization |  |
| --- | --- | --- | --- | --- |
| Si source | B1 | B2 | B1 | B2 |
| **NBRIP+MgT(0.5%)** | **26.63±6.38** | **37.50±5.25** | **93.28±3.58** | **64.03±45.17** |
| NBRIP+Talc(0.5%) | 0.00±0.00 | 0.00±0.00 | 17.55±7.80 | 173.23±20.48 |
| NBRIP+Feldspar(0.5%) | 382.13±8.63 | 80.25±17.25 | 368.88±80.28 | 264.55±43.55 |
| P-source | B1 | B2 | B1 | B2 |
| NBRISi*+KH_2_PO_4_(0.5%)-TCP | 0.00±0.00 | 0.00±0.00 | 48.43±0.33 | 32.50±8.45 |
| NBRISi+TCP(0.5%) | 47.25±1.50 | 53.25±4.50 | 37.38±1.63 | 29.58±2.93 |
| NBRISi+SP(0.5%)-TCP | 76.88±4.88 | 0.00±0.00 | 36.08±0.98 | 12.03±11.38 |
| NBRISi+HA(0.5%)-TCP | 85.88±6.38 | 76.88±6.38 | 4.23±1.63 | 0.00±0.00 |
| **NBRISi+HA(0.25%)-TCP** | **45.38±7.88** | **73.50±10.50** | **4.55±0.00** | **0.00±0.00** |
| NBRISi+HA(1.00%)-TCP | 55.13±5.63 | 51.38±1.88 | 10.40±0.00 | 0.98±0.33 |

***NBRISi: NBRIP+MgT(0.5%),** where, NBRIP contains l^-1^:10 g glucose; 5 g Ca_3_(PO_4_)_2_; 5 g MgCl_2_.6H_2_O; 0.25 g MgSO_4_.7H_2_O; 0.2 g KCl and 0.1 g (NH_4_)_2_SO_4_.). Changes in the NBRIP components are indicated within brackets. The data are means of three experiments.

MgT- magnesium trisilicate, HA- hydroxyapatite, TCP- tri-Calcium phosphate, SP- sodium phytate

Table S2 Effect of different carbon and nitrogen sources on silicon and phosphate solubilization by bacterial strains [B1 (*Bacillus safensis*) and B2 (*Bacillus amyloliquefaciens*)] in NBRIP broth media

| **Carbon Source** | | | | |
| --- | --- | --- | --- | --- |
| Treatments | **Si solubilization** | | **P solubilization** | |
|  | **B1** | **B2** | **B1** | **B2** |
| NBRISiH* +SC(1%)-Glu | 0.00±0.00 | 6.00±3.00 | 1.95±0.00 | 34.45±0.00 |
| NBRISiH +SA(1%)-Glu | 0.00±0.00 | 0.00±0.00 | 12.03±2.28 | 0.00±0.00 |
| NBRISiH +Sorbitol(1%)-Glu | 13.13±6.38 | 1.13±0.38 | 1.63±0.33 | 0.00±0.00 |
| NBRISiH +SB(1%)-Glu | 49.88±1.13 | 0.00±0.00 | 9.43±0.33 | 49.40±0.65 |
| NBRISiH +Sucrose(1%)-Glu | 64.88±1.13 | 10.88±2.63 | 48.43±0.33 | 39.98±0.33 |
| NBRISiH +Lactose(1%)-Glu | 50.63±6.38 | 12.00±4.50 | 7.80±0.00 | 22.43±1.63 |
| NBRISiH +Mannitol(1%)-Glu | 49.50±6.00 | 9.00±1.50 | 6.83±1.63 | 0.00±0.00 |
| NBRISiH +L-arabinose(1%)-Glu | 102.75±0.75 | 87.38±1.13 | 10.08±2.28 | 0.00±0.00 |
| NBRISiH +Arabinose(0.5%)-Glu | 82.88±0.38 | 52.13±2.63 | 6.50±0.00 | 22.10±1.95 |
| NBRISiH +Arabinose(1.5%)-Glu | 115.50±3.75 | 83.63±2.63 | 33.80±0.65 | 11.05±1.95 |
| **NBRISiH +Glu(0.25%)** | **180.00±5.25** | **323.63±28.88** | **1.95±0.65** | **6.83±1.63** |
| NBRISiH +Glu(0.5%) | 193.13±1.13 | 471.38±12.38 | 6.18±2.93 | 6.18±0.98 |
| NBRISiH +Glu(1%) | 212.25±19.50 | 516.75±17.25 | 0.00±0.00 | 10.73±0.98 |
| NBRISiH +Glu(1.5%) | 320.63±19.13 | 526.88±18.38 | 0.00±0.00 | 17.23±3.58 |
| **Nitrogen Source** | | | | |
| NBRISiHG +AFC (0.01%)-AS | 265.13±38.63 | 392.63±12.38 | 13.13±4.88 | 12.38±2.63 |
| NBRISiHG +AT (0.01%)AS | 1075.50±12.00 | 741.38±7.13 | 17.25±2.25 | 17.25±2.25 |
| NBRISiHG +AlAS (0.01%)-AS | 523.50±1.50 | 195.00±0.75 | 10.13±2.63 | 10.13±2.63 |
| NBRISiHG +AC (0.005%)-AS | 172.88±8.63 | 606.00±0.00 | 9.38±0.38 | 9.38±0.38 |
| NBRISiHG +AC (0.01%)-AS | 45.00±15.75 | 672.75±24.75 | 1.13±0.38 | 10.50±3.00 |
| NBRISiHG +AC (0.02%)-AS | 172.50±19.50 | 820.50±31.50 | 2.25±0.75 | 9.75±1.50 |
| NBRISiHG +AS (0.005%) | 147.38±12.38 | 242.25±21.75 | 3.00±0.75 | 1.50±0.00 |
| **NBRISiHG +AS (0.01%)** | 191.25±13.50 | 292.88±4.13 | 1.50±0.00 | 3.38±0.38 |
| NBRISiHG +AS(0.02%) | 284.25±29.25 | 391.50±81.75 | 2.63±0.38 | 1.88±0.38 |

*** NBRISiH- NBRISi+HA(0.25%), where,** NBRISiH contains l^-1^: 5 g MgT; 10 g glucose; 2.5 g HA; 5 g MgCl_2_.6H_2_O; 0.25 g MgSO_4_.7H_2_O; 0.2 g KCl and 0.1 g (NH_4_)_2_SO_4_)

****NBRISiHG- NBRISiH+Glu(0.25%),** **where,** NBRISiH contains l^-1^: 5 g MgT; 2.5 g glucose; 2.5 g HA; 5 g MgCl_2_.6H_2_O; 0.25 g MgSO_4_.7H_2_O; 0.2 g KCl and 0.1 g (NH_4_)_2_SO_4_.). Changes in the NBRISiH and NBRISiHG components are indicated within brackets. The data are means of three experiments.

MgT- magnesium trisilicate, Un C- uninoculated control, HA- hydroxyapatite, Glu- glucose, SC- sodium citrate, SA- sodium acetate, SB- sodium benzoate, AN- ammonium nitrate, ammonium sulphate, AFC- ammonium ferric citrate, AT- ammonium tartarate, AlAS- aluminium ammonium sulphate, AC- ammonium chloride.

Table S3 Effect of different salt sources on silicon and phosphate solubilization by bacterial strains [B1 (*Bacillus safensis*) and B2 (*Bacillus amyloliquefaciens*)] in NBRIP broth media

|  | **Si solubilization** | | **P solubilization** | |
| --- | --- | --- | --- | --- |
| **Treatments** | **B1** | **B2** | **B1** | **B2** |
| NBRISS +K_2_CO_3_(0.25%)-MgSO_4_-MgCl_2_-KCl | 157.13±16.13 | 261.38±11.63 | 14.63±4.13 | 0.00±0.00 |
| NBRISS +MgSO_4_(0.25%)-MgSO_4_-MgCl_2_-KCl | 456.00±60.00 | 345.00±36.75 | 5.25±3.75 | 0.00±0.00 |
| NBRISS +KCl(0.25%)-MgSO_4_-MgCl_2_-KCl | 395.63±57.38 | 315.00±30.00 | 0.00±0.00 | 0.00±0.00 |
| NBRISS +K_2_SO_4_(0.25%)-MgSO_4_-MgCl_2_-KCl | 413.25±7.50 | 312.00±7.50 | 4.88±0.38 | 0.00±0.00 |
| NBRISS +CaSO_4_(0.25%)-MgSO_4_-MgCl_2_-KCl | 784.13±85.13 | 317.25±25.50 | 0.00±0.00 | 0.00±0.00 |
| NBRISS +Na_2_CO_3_(0.25%)-MgSO_4_-MgCl_2_-KCl | 183.38±13.13 | 3.38±11.63 | 12.38±0.38 | 0.00±0.00 |
| NBRISS +NaCl(0.25%)-MgSO_4_-MgCl_2_-KCl | 283.13±13.88 | 140.25±62.25 | 0.00±0.00 | 0.00±0.00 |
| NBRISS +Na_2_SO_4_(0.25%)-MgSO_4_-MgCl_2_-KCl | 295.88±6.38 | 319.50±16.50 | 6.00±3.00 | 0.00±0.00 |
| NBRISS +NaNo_3_(0.25%)-MgSO_4_-MgCl_2_-KCl | 341.63±10.13 | 379.50±0.75 | 2.25±0.00 | 0.00±0.00 |
| NBRISS +CaCl_2_(0.25%)-MgSO_4_-MgCl_2_-KCl | 841.13±41.63 | 387.75±3.00 | 7.13±1.13 | 0.00±0.00 |
| NBRISS +MgCl_2_(0.25%)-MgSO_4_-MgCl_2_-KCl | 352.13±21.38 | 283.88±54.38 | 13.13±4.13 | 0.00±0.00 |
| NBRISS +Mg(NO_3_)_2_(0.25%)-MgSO_4_-MgCl_2_-KCl | 438.38±27.38 | 320.25±27.00 | 5.25±0.75 | 0.00±0.00 |
| NBRISS +CaCO_3_(0.25%)-MgSO_4_-MgCl_2_-KCl | 229.50±39.00 | 330.75±7.50 | 0.00±0.00 | 0.00±0.00 |
| NBRISS +Ca(NO_3_)_2_(0.25%)-MgSO_4_- MgCl_2_-KCl | 589.88±61.88 | 385.88±13.88 | 0.00±0.00 | 0.00±0.00 |
| NBRISS +KNO_3_(0.25%)-MgSO_4_-MgCl_2_-KCl | 396.38±40.88 | 414.38±33.38 | 8.63±1.88 | 0.00±0.00 |
| NBRISS +Mg(NO_3_)_2_(0.25%) +CaCl_2_(0.25%)-MgSO_4_-MgCl_2_-KCl | 496.50±6.75 | 696.75±17.25 | 13.88±1.88 | 0.00±0.00 |
| NBRISS +Mg(NO_3_)_2_(0.25%) +CaCl_2_(0.125%)-MgSO_4_-MgCl_2_-KCl | 318.38±7.13 | 409.88±19.88 | 8.25±0.00 | 0.00±0.00 |
| NBRISS +Mg(NO_3_)_2_(0.25%) +CaCl_2_(0.5%)-MgSO_4_-MgCl_2_-KCl | 628.50±3.75 | 930.00±12.00 | 14.25±2.25 | 0.00±0.00 |
| NBRISS +Mg(NO_3_)_2_(0.125 %)+ CaCl_2_(0.125%)-MgSO_4_-MgCl_2_-KCl | 495.38±10.13 | 741.38±27.38 | 2.25±0.75 | 0.00±0.00 |
| NBRISS +Mg(NO_3_)_2_(0.125 %)+ CaCl_2_(0.25%)-MgSO_4_-MgCl_2_-KCl | 830.63±11.63 | 724.88±25.13 | 8.25±1.50 | 0.00±0.00 |
| NBRISS +Mg(NO_3_)_2_(0.125 %)+ CaCl_2_(0.5%)-MgSO_4_-MgCl_2_-KCl | 705.38±10.13 | 617.25±8.25 | 16.13±4.88 | 0.00±0.00 |
| NBRISS +Mg(NO_3_)_2_(0.5 %)+ CaCl_2_(0.125%)-MgSO_4_-MgCl_2_-KCl | 587.63±10.88 | 468.75±4.50 | 0.00±0.00 | 0.00±0.00 |
| NBRISS +Mg(NO_3_)_2_(0.5 %)+ CaCl_2_(0.25%)-MgSO_4_-MgCl_2_-KCl | 658.50±6.75 | 773.63±3.38 | 9.75±0.00 | 0.00±0.00 |
| NBRISS +Mg(NO_3_)_2_(0.5 %)+ CaCl_2_(0.5%)-MgSO_4_-MgCl_2_-KCl | 572.63±0.38 | 1035.00±18.75 | 3.75±0.75 | 0.00±0.00 |

***** **NBRISS- NBRISiHG +AS(0.01%), where,** NBRISS contains l^-1^: 5 g MgT; 2.5 g glucose; 2.5 g HA; 5 g MgCl_2_.6H_2_O; 0.25 g MgSO_4_.7H_2_O; 0.2 g KCl and 0.1 g (NH_4_)_2_SO_4_.)

****NBRISSM** (final designed media): contains l^-1^: 2.5 g glucose; 2.5 g hydroxyapatite; 1.25 g MgNO_3_; 1.25 g CaCl_2_; 0.1 g (NH_4_)_2_SO_4_; 5 g Mg_2_O_8_Si_3_). Changes in the NBRISS components are indicated within brackets. The data are means of three experiments.

**
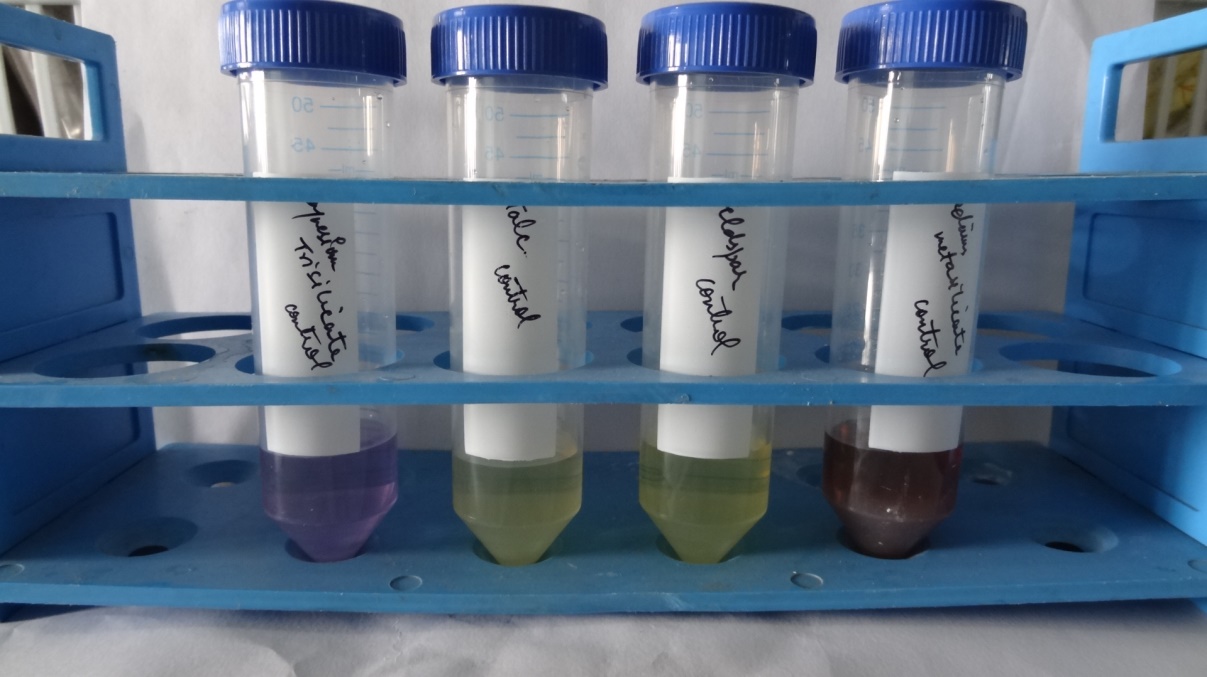
**

**a b c**

**Fig. S1** Selection of magnesium tri-silicate as silicon source in NBRISSM media for qualitative screening (a-magnesium trisilicate, b- talc, c- feldspar)

**Fig. S2** Shift in absorption maxima of Bromocresol Purple (BCP) at different concentrations *viz*. 0.00125%, 0.0025%, 0.005% and 0.01%

**Fig. S3** Comparative quantitative estimation of pH dependent phenomenon of Si solubilizers. P-solubilization (both in NBRIP media [a] and NBRISSM media [b]); Si-solubilization (c), Acidic phosphatase (d), alkaline phosphatase (e) gluconic acid (f), tartaric acid (g), maleic acid (h), succinic acid (i) and fumaric acid (j) (all in NBRISSM media).

Table S4 Si content of soil representing different locations and rhizospheres

| **S. No.** | **Location** | **Latitude** | **Sites** | **Rhizospheric soil of different crops** | **Soluble Si (mg/Kg)** |
| --- | --- | --- | --- | --- | --- |
| 1 | **U- Uttar Pradesh**  (i) B- Bulandshahr  (ii) D- Devkhera | 28.4070° N, 77.8498° E  27.1592° N, 78.3957° E | 8 (Maukhera, Mohammadpur, Kurval Banaras, Khera, Maukhera, Kamalpur, Gautam budh nagar, Akbarpur)  2 | Maize, Sugarcane, Seasame, Jowar, Mustard, Bajra, Chickpea  Rice | 28-58.65  00.00 |
| 2 | P- **Punjab** | 31.1471° N, 75.3412° E | 2 (Fazilka, Moga) | Rice | 15-17.25 |
| 3 | H- **Haryana** | 29.0588° N, 76.0856° E | 1 (Sirsa) | Rice | 44.10 |
| 4 | G- **Gujarat** | 22.2587° N, 71.1924° E | 1 (Morbi) | Cotton | 64.50 |

Table S5 *Invitro* screening of biocontrol agents against *R.solani*

| **S. no.** | **Strains** | **Antifungal activity against**  ***Rhizoctonia solani*** | **Control (C)**  **(mm)** | **Bacteria (T)**  **(mm)** | **Zone of inhibition I%=[(C-T)/C]x100** |
| --- | --- | --- | --- | --- | --- |
| 1 | vs9 | ++ | 1.8 | 1.1 | 38.88 |
| 2 | vs16 | + | 1.8 | 1.45 | 19.44 |
| 3 | vs1 | +++ | 1.8 | 0.8 | 55.55 |
| 4 | vs5 | - | 1.8 | 2.3 | -27.77 |
| 5 | vs8 | - | 1.8 | 2.5 | -38.88 |
| 6 | vs14 | ++ | 4.0 | 2.5 | 37.50 |
| 7 | vs6 | + | 1.8 | 1.5 | 16.66 |
| 8 | vs4 | ++ | 4.0 | 2.1 | 47.50 |
| 9 | vs12 | - | 1.8 | 1.8 | 0 |
| 10 | vs10 | - | 1.8 | 2.5 | -38.88 |
| 11 | vs7 | - | 1.8 | 2.5 | -38.88 |
| 12 | vs3 | - | 1.8 | 2.6 | -44.44 |
| 13 | sp5 | - | 1.8 | 1.8 | 0 |

+/- indicates the antifungal efficiency of bacterial strains against *Rhizoctonia solani*


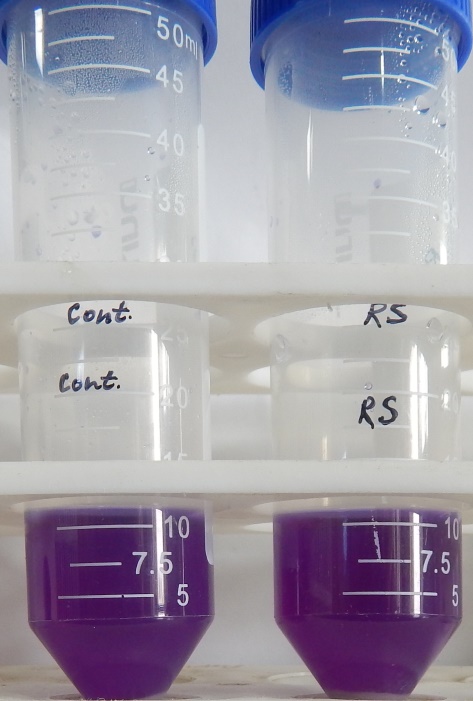


**a**

**b**

**Fig S4 :** Silicon solubilization by *R. solani* (RS) in NBRISSM media. Uninoculated (a), RS inoculated (b).

**A**

**B**

**Fig. S5** [A] growth inhibition assay of *R.solani* using identified metabolic components [B] % growth inhibition

Table S6 Metabolic components identified in ethyl acetate extract during *invitro* interaction of *Bacillus amyloliquefaciens* and *Rhizoctonia solani* (SN13-RS)

| **S.No.** | **Code used in PCA** | **Metabolites** | **Nature of compound** | **Peak area %** | | | **Functions** | **References** |
| --- | --- | --- | --- | --- | --- | --- | --- | --- |
|  |  |  |  | **SN** | **RS** | **S+R** |  |  |
| 1 | T1 | 5-Methoxymethyl-[1,3,4]thiadiazol-2-ylamine | heterocyclic compound | 0.76 | 0 | 1.09 | antimicrobial (fungicidal) activity | Chu et al., 2002; Amir et al., 2009; Serban et al., 2018 |
| 2 | T3 | 1H-Imidazole | aromatic heterocycle | 3.02 | 23.41 | 6.57 | ROS inhibitors or NADPH oxidase inhibitors | Lu and Yao, 2018 |
| 3 | T4 | N-Acetyl-L-glutamic acid | Amino acid | 1.32 | 1.19 | 1.23 | amino acid metabolism in plants; precursor for chlorophyll synthesis in developing leaves; precursor for both proline and ornithine biosynthesis in plants | Forde and Lea, 2007 |
| 4 | T5 | (+)-3,4-Dehydroproline amide | amide | 0.38 | 1.17 | 0.26 | inhibits biosynthesis of Hydroxyproline rich glycoproteins (inhibits proline hydroxylation) | Xu et al., 2011 |
| 5 | T6 | 1-O-hexadecylglycerol - bis-trimethylsilyl ether derivative | fatty alcohol | 1.46 | 0.77 | 0.53 | ether lipid precusor for its biosynthesis | Dittrich-Domergue et al., 2014 |
| 6 | T7 | Butanedioic acid | dicarboxylic acid | 1.05 | 0 | 0 | signal molecule during host-pathogen interactions; contributes to plant growth; increase mycotoxin production and activity of pathogenic enzymes causing increased virulence and pathogenesis to plants | Wu et al., 2011 |
| 7 | T9 | Benzoic acid, 4-ethoxy-, ethyl ester | Aromatic acid ester | 0.49 | 0.23 | 0.33 | antioxidant and free radical scavenging properties with promising antibacterial activities against the test pathogens | Arokiyaraj et al., 2018 |
| 8 | T10 | D-Fructose, 1,3,4,5,6-pentakis-O-(trimethylsilyl)-, O-methyloxime | Ketonic monosaccharide | 6.64 | 3.25 | 6.82 | Involved in stress mediated signalling | Srivastava et al., 2016 |
| 9 | T11 | d-Glucose, 2,3,4,5,6-pentakis-O-(trimethylsilyl)-, o-methyloxyme, (1E)- | carbohydrate | 6.26 | 2.2 | 6.33 | High glucose is associated with toxicity and pathogenesis through increased production of ROS by glucose auto oxidation and glucose metabolism | Srivastava et al., 2016 |
| 10 | T12 | Clarithromycin | azalide | 0.65 | 0 | 0 | antimicrobial activity | Das et al., 2016 |
| 11 | T13 | Xylulose, o-methyloxime, tetrakis-O-(trimethylsilyl)- | carbohydrate | 0.83 | 0 | 0.7 | biosynthesis of plastidial isoprenoids (essential for plant growth) | Hemmerlin et al., 2006; joyard et al., 2009 |
| 12 | T18 | Heptacosane | straight chain alkane | 0 | 0.58 | 0 | antibacterial activity | Konovalova et al., 2013 |
| 13 | T20 | n-Hexadecanoic acid | saturated fatty acid | 0.86 | 0.86 | 2.33 | decrease membrane fluidity | Zhukov, 2015 |
| 14 | T21 | 1,2-Benzenedicarboxylic acid, bis(2-methylpropyl) ester | ester | 0.25 | 0.7 | 0.44 | antimicrobial activity | Mohamad et al., 2018 |
| 15 | T23 | Dibutyl phthalate | diester | 2.59 | 6.4 | 12.96 | antifungal activity; bioactive defense factor | Nandhini et al., 2015; Ahsan et al., 2017; Kumar and Srimeena, 2019 |
| 16 | T27 | Pyrrolo[1,2-a]pyrazine-1,4-dione, hexahydro-3-(2-methylpropyl)- | heterocyclic compound | 2.98 | 2.08 | 0.5 | antibacterial and antifungal metabolite; antioxidant agent | Sathiyanarayanan et al., 2014; Sharma et al., 2014 |
| 17 | T28 | Diphenyl sulfone | organosulfur compound | 0 | 0 | 0.69 | induce plant immune responses | Schreiber et al., 2008 |
| 18 | T30 | Octadecanoic acid, 2,3-bis[(trimethylsilyl)oxy]propyl ester | saturated fatty acid | 0 | 1.03 | 0.47 | antibacterial activity | Sowbaraniga and Chitra, 2019 |
| 19 | T31 | Benzene, 1,1'-[3-(2-phenylethylidene)-1,5-pentanediyl]bis- | aromatic hydrocarbon | 0 | 0.52 | 0 | protection against biotic and abiotic stresses | Bhattacharya et al., 2010 |
| 20 | T33 | Ergotaman-3',6',18-trione, 9,10-dihydro-12'-hydroxy-2'-methyl-5'-(phenylmethyl)-, (5'α,10α)- | alkaloid | 0 | 0.84 | 0 | ergot alkaloids produced by the fungus genus Claviceps, which lives on cereal kernels and grass seeds. | Miedaner, T. and Geiger, H.H., 2015 |
| 21 | T34 | 13-Docosenamide, (Z)- | fatty acid amide | 9.75 | 4.75 | 6.3 | anti-microbial activity; probable role in host-bacterial relationships and host-pathogen interactions | Sharmila et al., 2017 |


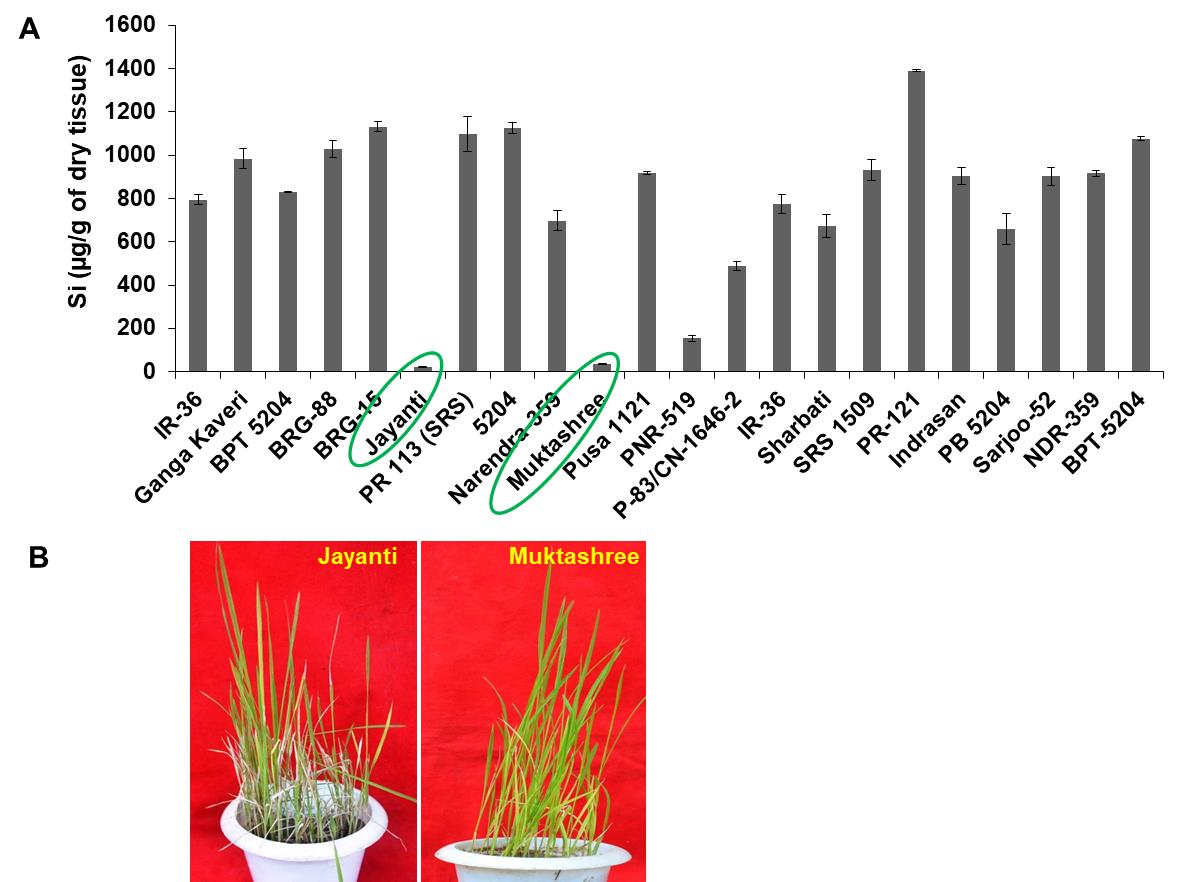


**Fig. S6** Silicon (Si) content of screened rice varieties (A); Disease susceptibility of rice varieties with lowest Si content (B).

**A**


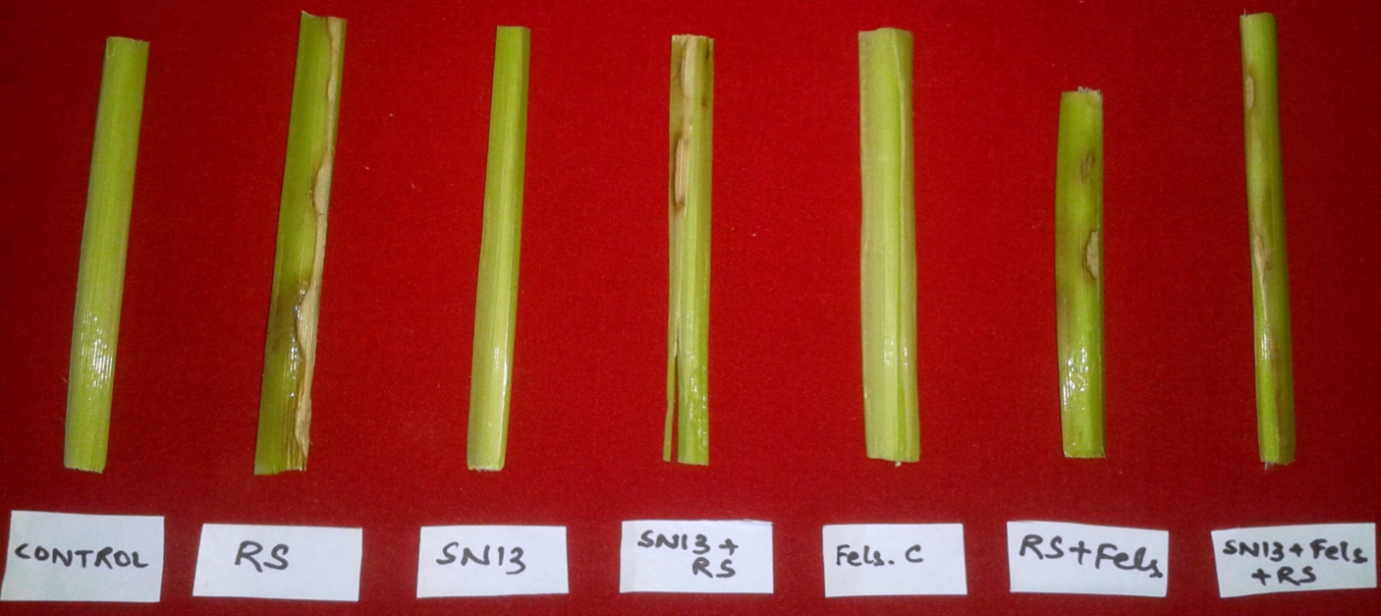


**0 4 0 3 0 3 2**

**B**

**
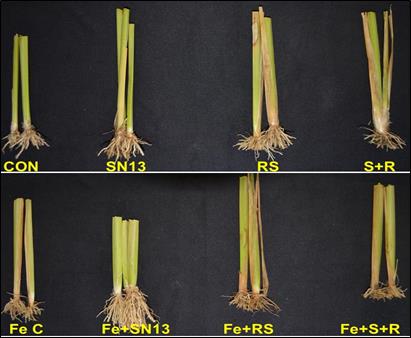
**

**Fig. S7** Disease severity in rice shoots at 15 day post infection of *R. solani*. Degree of disease severity (A): 0 = no lesion, 1 = appearance of water-soaked lesion, 2 = appearance of necrotic lesion, 3 = less than 50% necrosis on the leaf, 4 = more than 50% necrosis on the leaf cross section, and 5 = necrosis in entire leaf section resulting in leaf death; emergence of disease from lower leaf sheaths (B).

Table S7 List of primers used for real time validation of selected genes

| **Category** | **Gene name** | **LOC No.** | **Primer sequence** |
| --- | --- | --- | --- |
| Defense | Phospholipase D | Os06g40180 | For: CACCTG AACGTCAATG GGCAG  Rev: TCTCCGGACACACTCCAGGC |
|  | Peroxidase precursor | Os01g73200 | For: CGACCCCACCATGGACAAGTG  Rev: TGGCGGTTTTGCAGGTCGACG |
|  | Glutathione S-transferase | Os10g38590 | For: GACGAGCTCATGAAGCAGACGC  Rev: CAGCACGACGTCGACGTAGCCG |
|  | PR10 family protein | Os12g36830 | For: TGGAAGGTCTTCTCCGACGCGC  Rev: ATCATCCACAGCAGGGTTGAGC |
|  | Subtilisin homologue | Os01g58280 | For: GGTCTCATGCTGCTCTCAAATC  Rev: AGGATTTATGTTCCCTCCTCC |
| Hormone | Oxidoreductase, aldo/keto reductase family protein  (Auxin induced family protein) | Os04g26910 | For: TTGGAGCACTATCTGTGAAGCT  Rev: CCAAGTAGTGTTGGCCATCGC |
|  | Gibberelin 20-oxidase | Os01g66100 | For: TCGAACGGGAGGTATAAGAGCT  Rev: CTGCGGCGTGGCGGCGCTCG |
|  | Auxin response factor 9 | Os04g36054.1 | For:CTCCTACGAGGAGGCCAAGCTGC  Rev: AGGCATGATTGTCCAGGTCGAC |
| Cell wall | Glucan endo-1,3-beta-glucosidase-related | Os03g61780 | For: AACTCGATGTACGGCTCCGACA  Rev: ACACTTTCTCCTTGACATATGC |
|  | 4-alpha-glucanotransferase | Os07g43390 | For: TGGCGGTGGTTCTGACAATCC  Rev: AGTCTGCTTCTCTTCCTCTGG |
|  |  | OsActin | For: GAGTATGATGAGTCGGGTCCAG  Rev: ACACCAACAATCCCAAACAGAG |

**REFERENCES**

1. Ahsan, T., Chen, J., Zhao, X., Irfan, M. and Wu, Y., 2017. Extraction and identification of bioactive compounds (eicosane and dibutyl phthalate) produced by Streptomyces strain KX852460 for the biological control of Rhizoctonia solani AG-3 strain KX852461 to control target spot disease in tobacco leaf. AMB Express, 7(1), p.54;
2. Amir M, Kumar A, Ali I, Khan SA. Synthesis of pharmaceutically important 1,3,4-thiadiazole and imidazolinone derivatives as antimicrobials. Indian J Chem. 2009
3. Arokiyaraj, S., Bharanidharan, R., Agastian, P. and Shin, H., 2018. Chemical composition, antioxidant activity and antibacterial mechanism of action from Marsilea minuta leaf hexane: methanol extract. *Chemistry Central Journal*, *12*(1), p.105.
4. Bhattacharya, A., Sood, P. and Citovsky, V., 2010. The roles of plant phenolics in defence and communication during Agrobacterium and Rhizobium infection. *Molecular plant pathology*, *11*(5), pp.705-719.
5. Chu, C.H., Hui, X.P., Xu, P.F., Zhang, Z.Y., Li, Z.C. and Liao, R.A., 2002. Synthesis and antifungal activities of ω-(5-arylamino-1, 3, 4-thiadiazol-2-thio)-ω-(1H-1, 2, 4-triazol-1-yl) acetophenones;.
6. Das, M.C., Sandhu, P., Gupta, P., Rudrapaul, P., De, U.C., Tribedi, P., Akhter, Y. and Bhattacharjee, S., 2016. Attenuation of Pseudomonas aeruginosa biofilm formation by Vitexin: A combinatorial study with azithromycin and gentamicin. *Scientific reports*, *6*, p.23347.
7. Dittrich-Domergue, F., Joubès, J., Moreau, P., Lessire, R., Stymne, S. and Domergue, F., 2014. The bifunctional protein TtFARAT from Tetrahymena thermophila catalyzes the formation of both precursors required to initiate ether lipid biosynthesis. *Journal of Biological Chemistry*, *289*(32), pp.21984-21994.
8. Forde, B.G. and Lea, P.J., 2007. Glutamate in plants: metabolism, regulation, and signalling. *Journal of experimental botany*, *58*(9), pp.2339-2358; Winter, G., Todd, C.D., Trovato, M., Forlani, G. and Funck, D., 2015. Physiological implications of arginine metabolism in plants. Frontiers in plant science, 6, p.534.
9. Hemmerlin, A., Tritsch, D., Hartmann, M., Pacaud, K., Hoeffler, J.F., van Dorsselaer, A., Rohmer, M. and Bach, T.J., 2006. A cytosolic Arabidopsis D-xylulose kinase catalyzes the phosphorylation of 1-deoxy-D-xylulose into a precursor of the plastidial isoprenoid pathway. Plant physiology, 142(2), pp.441-457;
10. Joyard, J., Ferro, M., Masselon, C., Seigneurin-Berny, D., Salvi, D., Garin, J. and Rolland, N., 2009. Chloroplast proteomics and the compartmentation of plastidial isoprenoid biosynthetic pathways. *Molecular plant*, *2*(6), pp.1154-1180.
11. Konovalova, O., Gergel, E. and Herhel, V., 2013. GC-MS analysis of bioactive components of Shepherdia argentea (Pursh.) Nutt. from Ukrainian Flora. *The Pharma Innovation*, *2*(6, Part A), p.7.
12. Kumari, S.M.P. and Srimeena, N., 2019. Arbuscular Mycorrhizal Fungi (AMF) Induced Defense Factors against the Damping-off Disease Pathogen, Pythium aphanidermatum in Chilli (Capsicum annum). *Int. J. Curr. Microbiol. App. Sci*, *8*(6), pp.2243-2248.
13. Lu, Y. and Yao, J. (2018). Chloroplasts at the Crossroad of Photosynthesis, Pathogen Infection and Plant Defense. Int. J. Mol. Sci. 19, 3900.
14. Miedaner, T. and Geiger, H.H., 2015. Biology, genetics, and management of ergot (Claviceps spp.) in rye, sorghum, and pearl millet. Toxins, 7(3), pp.659-678.
15. MOHAMAD, A., ABDALLA, O., Li, L., Ma, J., Hatab, S.R., Xu, L., Guo, J.W., Rasulov, B.A., Liu, Y.H., Hedlund, B.P. and Li, W.J., 2018. Evaluation of the antimicrobial activity of endophytic bacterial populations from Chinese traditional medicinal plant licorice and characterization of the bioactive secondary metabolites produced by Bacillus atrophaeus against Verticillium dahliae. *Frontiers in Microbiology*, *9*, p.924.
16. Nandhini, U.S., Sangareshwari, S. and Lata, K., 2015. Gas chromatography-mass spectrometry analysis of bioactive constituents from the marine Streptomyces. Asian Journal of Pharmaceutical and Clinical Research, 8(2), pp.244-246;
17. Sathiyanarayanan, G., Gandhimathi, R., Sabarathnam, B., Kiran, G.S. and Selvin, J., 2014. Optimization and production of pyrrolidone antimicrobial agent from marine sponge-associated Streptomyces sp. MAPS15. *Bioprocess and biosystems engineering*, *37*(3), pp.561-573
18. Schreiber, K., Ckurshumova, W., Peek, J. and Desveaux, D., 2008. A high‐throughput chemical screen for resistance to Pseudomonas syringae in Arabidopsis. The Plant Journal, 54(3), pp.522-531; Bektas, Y. and Eulgem, T., 2015. Synthetic plant defense elicitors. *Frontiers in plant science*, *5*, p.804.
19. Serban, G., Stanasel, O., Serban, E. and Bota, S., 2018. 2-Amino-1, 3, 4-thiadiazole as a potential scaffold for promising antimicrobial agents. Drug design, development and therapy, 12, p.1545.
20. Sharma, P., Kalita, M.C. and Thakur, D., 2016. Broad spectrum antimicrobial activity of forest-derived soil actinomycete, Nocardia sp. PB-52. Frontiers in microbiology, 7, p.347.
21. Sharmila, M., Rajeswari, M. and Jayashree, I., 2017. GC-MS Analysis of Bioactive Compounds in the Whole Plant of Ethanolic Extract of Ludwigiaperennis L. *Int J Pharm Sci Rev Res*, *46*(1), pp.124-128.
22. Sowbaraniga, R. and Chitra, M., 2019. Phytocompounds identification in Cucumis dipsaceus Ehrenb ex. Spach fruits by gas chromatography-mass spectrometry (GC-MS) analysis. *Journal of Pharmacognosy and Phytochemistry*, *8*(2), pp.1453-1458.
23. Srivastava, S., Bist, V., Srivastava, S., Singh, P.C., Trivedi, P.K., Asif, M.H., Chauhan, P.S. and Nautiyal, C.S. (2016). Unraveling aspects of Bacillus amyloliquefaciens mediumted enhanced production of rice under biotic stress of Rhizoctonia solani. Front. Plant. Sci. 7, 587. doi: 10.3389/fpls.2016.00587
24. Wu, H.S., Liu, Y.D., Zhao, G.M., Chen, X.Q., Yang, X.N. and Zhou, X.D., 2011. Succinic acid inhibited growth and pathogenicity of in vitro soil-borne fungus Fusarium oxysporum f. sp. niveum. *Acta Agriculturae Scandinavica, Section B-Soil & Plant Science*, *61*(5), pp.404-409.
25. Xu, C., Takáč, T., Burbach, C., Menzel, D. and Šamaj, J., 2011. Developmental localization and the role of hydroxyproline rich glycoproteins during somatic embryogenesis of banana (Musa spp. AAA). *BMC plant biology*, *11*(1), p.38.
26. Zhukov, A.V. (2015). Palmitic acid and its role in the structure and functions of plant cell membranes. Russ. J. Plant Physiol. 62, 706-713.
